# The relative impact of underweight, overweight, smoking, and physical inactivity on health and associated costs in Indonesia: propensity score matching of a national sample

**DOI:** 10.1186/s12913-022-08546-6

**Published:** 2022-09-17

**Authors:** Zulfikar Ihyauddin, Tiara Marthias, Kanya Anindya, Nawi Ng, Fatwa Sari Tetra Dewi, Emily S. G. Hulse, Reza Pandu Aji, Dwi Astuti Dharma Putri, John Tayu Lee

**Affiliations:** 1 Nossal Institute for Global Health, Melbourne School of Population and Global Health, The University of Melbourne, Level 4, 333 Exhibition St, Melbourne VIC 3004, Australia; 2 School of Public Health and Community Medicine, Institution of Medicine, Sahlgrenska Academy, University of Gothenburg, Gothenburg, Sweden; 3 Department of Health Behavior, Environment, and Social Medicine, Faculty of Medicine, Public Health, and Nursing, Universitas Gadjah Mada, Yogyakarta, Indonesia; 4 Centre for Health Policy, School of Population and Global Health, The University of Melbourne, Melbourne, VIC Australia; 5 Center for Child Health – Pediatric Research Office, Faculty of Medicine, Public Health, and Nursing, Universitas Gadjah Mada, Yogyakarta, Indonesia; 6 Department of Primary Care and Public Health, School of Public Health, Imperial College London, London, UK

**Keywords:** Tobacco use, Alcohol consumption, BMI, Non-communicable disease, Indonesia

## Abstract

**Background:**

Indonesia is in the middle of a rapid epidemiological transition with an ageing population and increasing exposure to risk factors for chronic conditions. This study examines the relative impacts of obesity, tobacco consumption, and physical inactivity, on non-communicable diseases multimorbidity, health service use, catastrophic health expenditure (CHE), and loss in employment productivity in Indonesia.

**Methods:**

Secondary analyses were conducted of cross-sectional data from adults aged ≥ 40 years (*n* = 12,081) in the Indonesian Family Life Survey 2014/2015. We used propensity score matching to assess the associations between behavioural risk factors and health service use, CHE, employment productivity, and multimorbidity.

**Results:**

Being obese, overweight and a former tobacco user was associated with a higher number of chronic conditions and multimorbidity (*p* < 0.05). Being a former tobacco user contributed to a higher number of outpatient and inpatient visits as well as CHE incidences and work absenteeism. Physical inactivity relatively increased the number of outpatient visits (30% increase, *p* < 0.05) and work absenteeism (21% increase, *P* < 0.06). Although being underweight was associated with an increased outpatient care utilisation (23% increase, *p* < 0.05), being overweight was negatively associated with CHE incidences (50% decrease, *p* < 0.05).

**Conclusion:**

Combined together, obesity, overweight, physical inactivity and tobacco use contributed to an increased number of NCDs as well as medical costs and productivity loss in Indonesia. Interventions addressing physical and behavioural risk factors are likely to have substantial benefits for individuals and the wider society in Indonesia.

**Supplementary Information:**

The online version contains supplementary material available at 10.1186/s12913-022-08546-6.

## Background

Indonesia, the fourth most populous country in the world, is in the midst of a rapid demographic and epidemiological transition. In 2016, non-communicable diseases (NCDs) were responsible for 73% of all deaths in this country, higher than its proportion in 2010, which was 64% [1, 2]. The prevalence of behavioural risk factors for NCDs has increased significantly over the past decade. For example, there has been an increase in the prevalence of obesity (from 15.4% in 2013 to 21.8% in 2018), tobacco smoking (from 27.2% in 1995 to 33.8% in 2018), and physical inactivity (from 26.1% in 2013 to 33.5% in 2018) [3–8]. However, many NCDs are preventable through public health interventions targeting behavioural change, such as increasing the pricing or regulation of tobacco and unhealthy foods or mass media campaigns to promote physical activity [9].

The United Nations High-level Meeting on NCDs in 2018 highlighted the enormous challenges of NCDs on health systems and wider society [10]. Since NCDs require long-term management, their treatment can lead to financial hardship. NCDs can also coexist and contribute to the growing burden of multimorbidity, which is defined as the presence of 2 or more NCDs [11]. Evidence from Indonesia and other low-middle income countries (LMICs) suggest that NCDs are also associated with substantial direct medical costs, i.e. the cost of seeking and utilising healthcare services, and indirect costs, i.e. the costs arising from productivity loss due to illness [12–15]. In Indonesia, the burden of direct costs of NCDs is reflected by the significant proportion of the costs under the national health insurance scheme [16]. In 2018, the eight diseases with the highest cost burden and complications in Indonesia were all NCDs and consumed up to 23% of the total service costs in referral health services (US$1.1 billion) under the national health insurance scheme [16]. The estimated indirect costs of NCDs in Indonesia are also substantial, accounting for approximately $4.47 trillion workplace productivity loss between 2012 and 2030 [12]. In addition, catastrophic health expenditure (CHE) also becomes a concern in Indonesia due to insufficient public spending on health. With only 3.2% allocation of the national budget to health, the proportion of out of pocket expenditure (OOPE) in Indonesia remained high at 31.8% in 2017 [17]. This is further aggravated by the considerable proportion of Indonesians who are at risk of experiencing CHE, particularly those living under the poverty line (10%) and having income close to the poverty line (37.3%) [18]. Therefore, failure to address and deal with NCD risk factors poses major barriers for Indonesia to achieve universal health coverage (UHC) as well as the 25*25 NCD targets set by the World Health Organization (WHO) [1, 19].

Understanding the relative impacts of behavioural risk factors on health outcomes and health care costs would guide priority setting for NCD prevention. Using a nationally-representative survey in Indonesia, we present the first study that investigates the relative impacts of several behavioural (e.g. tobacco use, physical inactivity) and metabolic risk factors (e.g. obesity) on NCDs, multimorbidity healthcare utilisation, catastrophic health expenditure, and loss in work productivity.

## Methods

### Data and sample

We used cross-sectional data from the 5th Wave of the Indonesian Family Life Survey (IFLS) collected in 2014/2015. In brief, the aim of the IFLS is to understand the health status, health care utilisation, health care costs of the adult population to inform evidence-based policy. The survey objectives and methods are detailed elsewhere [20]. We included a total of 12,081 respondents aged ≥ 40 years in this current study. Sample flowcharts of this study are presented in Figures S1-S4. Ethical approval for the IFLS study was obtained from the ethics review boards of RAND and Gadjah Mada University in Indonesia.

### Variables

#### Independent variables

The independent variables were age, body mass index (BMI), tobacco consumption, and physical activity. Information on age was obtained by asking “How old are you?” and divided into four categories, i.e. 40–49, 50–59, 60–69, and ≥ 70 years old. BMI was calculated using weight and height, and was defined according to the WHO’s Asian-Pacific cut-off points, i.e. < 18.5 kg/m2 (underweight), 18.5–22.9 kg/m2 (normal weight), 23–24.9 kg/m2 (overweight), and ≥ 25 kg/m2 (obesity) [21, 22]. Tobacco consumption was assessed using two questions: 1) “Have you ever chewed tobacco, smoked a pipe, smoked self-enrolled cigarettes, or smoked cigarettes/cigars?”; and 2) “Do you still have the habit or have you totally quit?”. Based on their responses, we categorised the respondents into never users, former tobacco users, light users (smoke 1–9 cigarettes/day or consume < 100 g tobacco/week), moderate users (smoke 10–19 cigarettes/day or consume 100–299 g tobacco/week) and heavy users (smoke ≥ 20 cigarettes/day or consume ≥ 300 g tobacco/week) [23, 24]. Furthermore, physical activity was assessed using the International Physical Activity Questionnaire (IPAQ) scoring protocol [25, 26]. Respondents were categorised as having low, moderate, or high levels of physical activity.

#### Outcome variables

There are four main dependent variables of NCDs, healthcare use and financial burden as the proxy for direct costs, and loss in employment productivity for indirect costs. A history of NCDs was determined by asking “Have a doctor/paramedic/nurse/midwife ever told you that you had any of these conditions?”. There are 14 self-reported NCDs that an individual can have, including hypertension, diabetes, asthma, heart attack/coronary heart diseases, liver disease, stroke, cancer, arthritis/rheumatism, high cholesterol, prostate illness (for male respondents), kidney diseases (excluding malignancy), digestive diseases, mental illness, and memory-related diseases. The number of NCDs that the participant answered “yes” was calculated and grouped into two variables, i.e. the number of chronic conditions and presence of multimorbidity (yes/no).

To measure healthcare use, we utilised the respondent's answers on the frequency of outpatient visits in the last four weeks and inpatient visits in the last 12 months. The financial burden was measured by the proportion of total healthcare expenditure to total household expenditure and the proportion of total healthcare expenditure to total non-food expenditure. CHE was then defined using different thresholds, i.e. (1) total health expenditures exceeding 25% of total household expenditure; and (2) total health expenditures exceeding 40% of total non-food expenditure. The former threshold was used according to the Sustainable Development Goals indicators on universal health coverage financial protection (indicator 3.8.2) [27], while the latter threshold captured the households’ ability to pay for non-nutritional needs [28–30].

Productivity loss was measured based on two variables: labour force participation and the number of days of primary activity missed due to poor health. Labour force participation status was based on the participant's response to employment status at the time of the survey.

### Covariates

In order to control for confounding, several sociodemographic variables were included as covariates in the statistical analysis, i.e. sex, marital status, educational level, residency, the region of residency, ethnicity, covered by health insurance, occupation, and quintiles of household per capita expenditures (q1/lowest to q5/highest). Further details on covariate variables can be found in Table S2.

### Statistical analysis

We used propensity score matching (PSM) to examine the associations between behavioural risk factors and outcomes. This method was used to reduce selection bias that commonly emerged in observational studies. By balancing the distributions of the observed covariates between treatment and control groups, PSM constructs matching pairs between treatment and control groups [31–33]. After matching, each pair underwent three steps of PSM. The first step was to use a logit model to estimate the propensity score, i.e. the probability of a respondent to be assigned to the treatment group according to the matching variables. A complete set of the covariates variables was used for each “treatment vs control” pair (a total of 12 pairs in our study) in the same independent variable. Moreover, we also added the rest of the independent variables that were not used in the analysis as covariates. For example, in the PSM analysis for the older age group, we included BMI, tobacco consumption, and physical activity as well. We aimed to ensure that the only difference between the treatment and the control groups was only the variable of interest (either age, BMI, tobacco consumption, or physical activity), while the other observed covariates were matched.

Secondly, every individual in each group was matched based on their propensity score using *psmatch2* command (STATA 14.2). Three PSM algorithms, namely the nearest neighbour (NN) with 1-to-1 replacement, NN without replacement, and Kernel matching, were performed to explore which algorithm generated the most satisfactory balance in all covariates. Thirdly, *kmatch* command was used to generate the average treatment effect on the treated (ATT) to estimate the effects of the dependent variable between treatment and control groups. The calliper of bandwidth used in the analysis was equal to 0.2 of the standard deviation of the propensity score logit. Furthermore, bootstrapping with 200 times replications was conducted to improve the accuracy of estimating the standard error. We present estimates from the propensity score matching models for the ATT of each behavioural risk factor. Additionally, to improve interpretation, we calculated the percentage change relative to the control group (i.e. respondents without the risk factor) via dividing ATT by the mean of the outcome for the control group.

Lastly, we performed sensitivity analyses based on conventional regression models. In this analysis, we used logistic regression for bivariate outcome and linear regression or zero-inflated negative binomial (ZINB) regression for count outcome. ZINB was used for continuous count outcomes that have over-dispersion characteristics, such as health service use, CHE, and productivity loss [34]. The coefficients obtained from the logistic and ZINB regression were then exponentiated to obtain the adjusted odds ratio (AOR) and the incidence rate ratio (IRR), respectively.

## Results

### Sample characteristics

The summary of participant characteristics is reported in Table 1. The median age was 51 years (IQR 45–61 years), 53.4% were female, 80.6% were currently married, 40.2% reported no formal education, 76.9% resided in Java-Bali islands, 50.8% resided in an urban area, and 48.2% had any type of health insurance. The proportions of people that were current tobacco users, former tobacco users, and non-tobacco users were 33.1%, 7.0%, and 59.9%, respectively. The proportion of people with high, moderate, and low levels of physical activity were 33.4%, 28.4% and 38.2%, respectively. Meanwhile, the proportion of respondents who were underweight, had normal BMI, overweight, and obese were 10.6%, 36.8%, 16.9%, and 35.7%, respectively. In this study, Kernel matching was chosen, as this algorithm yielded sufficient output based on the t-test results on covariate balance check. Sample characteristics before matching for each level of the independent variables and the covariate balance check after matching, are presented in Table S2-S17.

**Table 1:** Characteristics of participants in the 5^th^ Wave of the Indonesian Family Life Survey (*n* = 12,081)

Characteristics	All (*n* = 12,081)^a^	
	%	n
Age group		
40–49 years	43.7	5237
50–59 years	30.2	3528
60–69 years	16.2	1967
70 + years	9.9	1349
Sex		
Female	53.4	6323
Male	46.6	5758
Marital status		
Not currently married	19.4	2455
Currently married	80.6	9626
Education		
No education	40.2	4747
Primary	25.6	2917
Junior High School	11.1	1387
Senior high school	16.7	2205
Tertiary	6.4	825
Ethnicity		
Javanese	56.2	5663
Sundanese	15.2	1475
Others	28.6	4943
Had any health insurance		
No	51.8	6055
Yes	48.2	5026
Type of work		
Unemployed	22.7	2845
Casual	17.7	2004
Self-employed	39.0	4720
Government/private	20.1	2512
Per capita consumption expenditure		
Q1 (the lowest)	21.3	2406
Q2	21.0	2425
Q3	19.9	2433
Q4	19.3	2410
Q5 (the highest)	18.5	2407
Residency		
Rural	49.2	5228
Urban	50.8	6853
Region		
Java-Bali	76.9	7586
Sumatra	15.0	2576
Nusa Tenggara	2.7	802
Kalimantan	2.7	550
Sulawesi	2.7	567
BMI (kg/m^2^)		
Underweight (< 18.5)	10.6	1303
Normal (18.5–23.0)	36.8	4410
Overweight (23.0- < 25.0)	16.9	2047
Obesity (≥ 25)	35.7	4321
Tobacco consumption		
Never use tobacco	59.9	7128
Former user	7.0	922
Light user	8.7	1051
Moderate user	19.1	2292
Heavy user	5.3	688
Physical activity		
High	33.4	3837
Moderate	28.4	3418
Low	38.2	4826
Number of NCDs		
0	37.9	4539
1	41.4	4953
2 +	20.7	2589

^a^Values are weighted percentages and unweighted counts

### The relative impacts of NCDs risk factors on chronic conditions

**Fig. 1:**
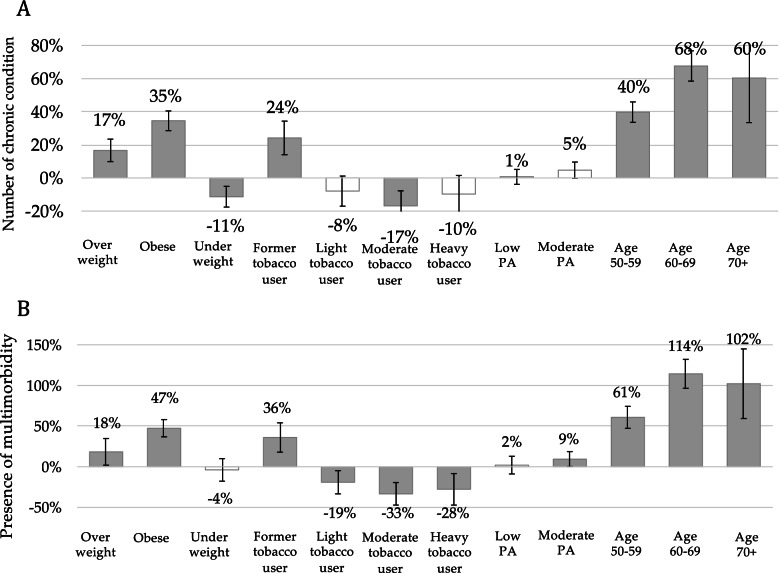
Relative impacts of NCDs risk factors on the number of chronic conditions and the presence of multimorbidity. Notes: Grey bar denotes significant association with *p*-value < 0.05

Regression results for NCDs and multimorbidity were presented in Fig. 1. We found that being overweight or obese was associated with an increased number of NCDs (17%, 95% CI 10%; 23% and 35%, 95% CI 29%; 40% increase, respectively) as well as an increase in multimorbidity (18% and 47% increase, respectively)), compared to having normal BMI. A moderate level of physical activity also increased the likelihood of multimorbidity by 9% (95% CI 0%; 19%), compared to being engaged in a high level of physical activity. However, we found no significant association between low physical activity and an increased number of NCDs or multimorbidity. Older age also contributed to an increase in the number of chronic conditions (60%, 95% CI 33%; 87% for age group ≥ 70 years) and multimorbidity (102%, 95% CI 57%; 143% increase for ≥ 70 years), compared to those in the age group 40–49 years. Other behavioural risk factors such as being a former tobacco user were associated with an increased number of chronic conditions (24%, 95% CI 14%; 35% increase) and the risk of multimorbidity (36%, 95% CI 16%; 52% increase), compared to being a non-smoker. On the other hand, being a moderate tobacco user was associated with a reduction in the number of chronic conditions by 17% (95% CI -27%; -8%) and multimorbidity by 33% (95% CI -44%; -17%), compared to those who never used tobacco. Another risk factor that had negative associations (Fig. 1) with the number of chronic conditions was being underweight (11%, 95% CI -18%; -6% decrease), compared to those with a normal BMI.

### The relative impacts of NCDs risk factors on health service use

**Fig. 2:**
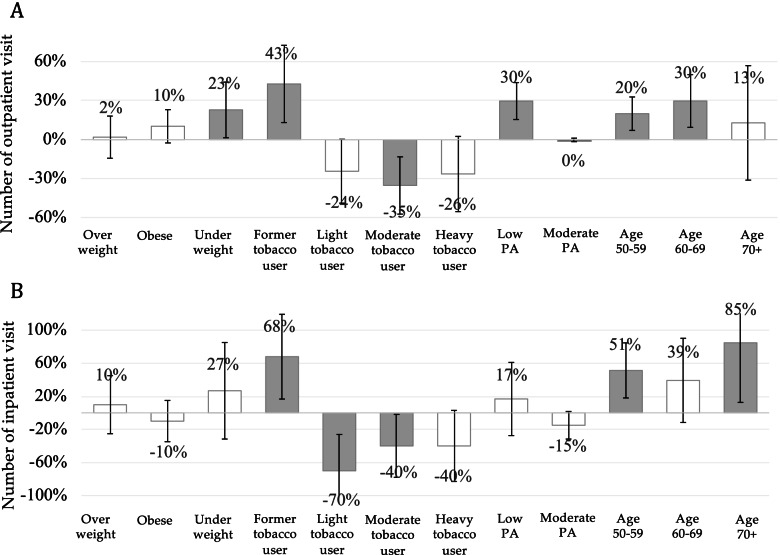
Relative impacts of NCDs risk factors on the number of outpatient and inpatient visits. Notes: Grey bar denotes significant association with *p*-value < 0.05

Results from regression analysis for health service use were presented in Fig. 2. The greatest contributor to an increased number of outpatient care in the last four weeks was being a former tobacco user (43%, 95% CI 12%; 71% increase), followed by having low physical activity (30%, 95% CI 15%; 44% increase), and being underweight (23%, 95% CI 3%; 46% increase). Being a former tobacco user was associated with an increased number of inpatient visits in the last 12 months (68%, 95% CI 13%; 115% increase). In contrast, being a moderate tobacco user was associated with a lower number of outpatient and inpatient visits (35%, 95% CI 59%; -15% and 40%, 95% CI 80%; -4% decrease, respectively), while being a light tobacco user was associated with 70% reduced number of inpatient visits (95% CI -105%; -18%).

### The relative impacts of NCDs risk factors on the financial burden

**Fig. 3:**
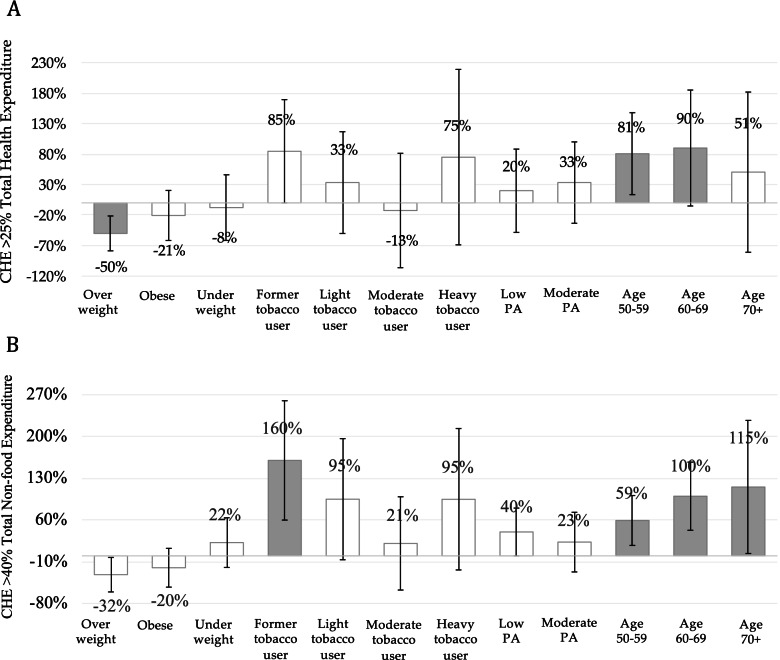
Relative impacts of NCDs risk factors on catastrophic health expenditure. Notes: Grey bar denotes significant association with *p*-value < 0.05

Results from the regression analysis for the financial burden are presented in Fig. 3. Being a former tobacco user was associated with an increased proportion of CHE, with health expenditure exceeding 40% of total non-food expenditure by 160% (95% CI 67%; 267%) compared to those who did not smoke. Meanwhile, being overweight was associated with reduced incidence of CHE for health expenditure exceeding 25% of total household expenditure by 50% (95% CI 71%; 14%), compared to those with normal BMI. Older age groups were also associated with the incidence of CHE for all thresholds.

### The relative impacts of NCDs risk factors on productivity loss

**Fig. 4:**
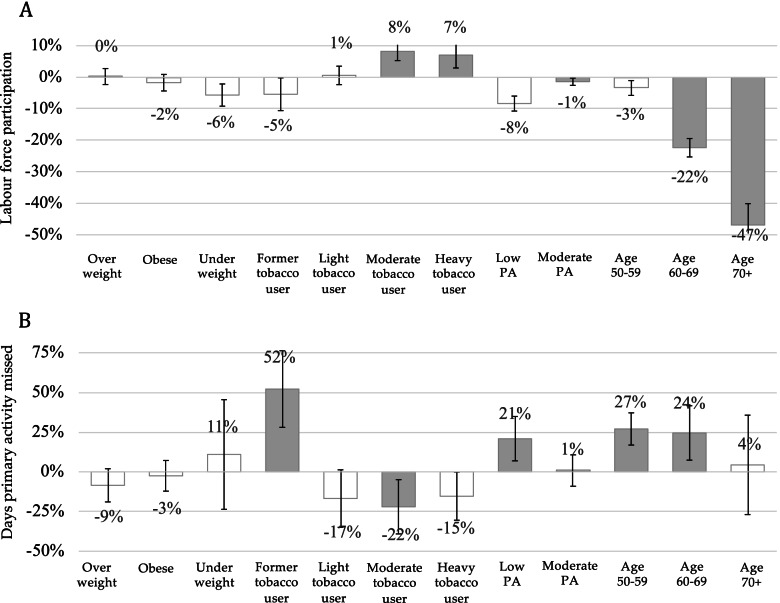
Relative impacts of NCDs risk factors on labour force participation and the number of days primary activity missed. Notes: Grey bar denotes significant association with *p*-value < 0.05

Results from regression analysis for productivity loss were presented in Fig. 4. The most significant contributor to reduced labour force participation was being older followed by moderate physical activity. Moderate physical activity was associated with reduced labour force participation by 1% (95% CI -3%; 0%), compared with respondents who did high physical activity. In contrast, moderate and heavy tobacco users were associated with a 8% (95% CI 5%; 11%) and 7% (95% CI 4%; 12%) likelihood of increased labour force participation, respectively, compared to non-users.

Productivity loss in terms of having a higher number of days of primary activity missed due to poor health was associated with former tobacco users, low physical activity, and older age groups. A 52% (95% CI 28%; 76%) relative increase in days of primary activity missed was observed among former tobacco users, compared to non-users. Meanwhile, among those who did low physical activity, the number of days primary activity missed were greater by 21% (95% CI 7%; 35%), compared to respondents who did high physical activity. A greater number of days of primary activity missed was also observed for the age group 50–59 and 60–69 years, accounting for a 27% (95% CI 17%; 37%) and 24% (95% CI 7%; 42%) increase, respectively, compared to age group 40–49 years old. In contrast, a smaller number of days of primary activity missed due to poor health was observed for moderate tobacco users, accounting for 22% (95% CI -39%; -5%) fewer days, compared to those who were non-tobacco users.

### Sensitivity analyses and robustness check

Our results from sensitivity analysis were consistent with those in the main analysis, as in most cases, the ATTs were similar across different matching algorithms. However, the Kernel algorithm showed some significant results that did not appear in other algorithms even though the direction of the ATTs were the same (see Table S18-S25). Moreover, the number of significant results from Kernel algorithm was more similar to NN with replacement algorithm, rather than NN without replacement. This could potentially emerge due to the comparable sample size between Kernel and NN with replacement algorithm, while in the NN without replacement, there was a higher number of participants that were off-support which might lead to imbalance covariates (Table S6-S17). Overall, comparable results were generated from PSM and the linear and ZINB regression, however some associations were significant in regression analysis but were not significant in the PSM approach (Table S26-S29).

## Discussion

### Summary of key findings

This study showed that being a former tobacco user, physically inactive and older significantly increased multimorbidity and costs related to health service use, OOPE, and employment productivity in Indonesia. Being overweight and obese were also associated with having an increased number of NCDs and multimorbidity.

This study shows that being older contributes significantly to higher rates of chronic conditions and multimorbidity, CHE, outpatient and inpatient care, work absenteeism, and lower labour force participation. This is unsurprising, considering that older age has been found to be a strong predictor of total societal and healthcare costs in other studies [35, 36]. Similar to previous studies conducted in Indonesia, our study reported that the prevalence of respondents who had at least 1 NCD increased with age (see Table S2) [37].

Physical inactivity is consistently associated with higher costs through greater outpatient service use (30%) and a greater number of days of primary activity missed (21%). Currently, there is a paucity of evidence on the impact of physical inactivity on health service use in Indonesia [38]. However, our results showed that physical inactivity was associated with a greater use of healthcare services, which are similar to previous findings from other LMICs [39]. For example, a study in South Africa revealed that respondents who were physically inactive were 24% more likely to use outpatient services [40]. Our results on productivity loss also corresponded with the existing evidence from high-income countries that find reverse associations between the level of physical activity and absenteeism among the working population [41–44]. However, it should be noted that the impact of physical activity to these outcomes is prone to reverse causation, of which having an illness might lead to lower physical activity, rather than the opposite.

Being overweight and obese increased the number of chronic conditions and the presence of multimorbidity. However, in contrast to previous evidence [40, 45–48], our results revealed that being overweight had a negative association towards the incidence of CHE. This association is most likely due to reverse causation which is common in cross-sectional study design, such as in the association between high BMI and mortality [49, 50]. In our study, reverse causation indicates that higher working productivity and CHE resilience precede the likelihood of having a high BMI. This was supported by the characteristics of respondents who were overweight (see Table S4), which were mostly in the productive age group (40–60 years, 80%) and participated in the labour market (75%). However, this association was not found in the obese population.

This study found that being a former tobacco user was associated with a greater number of chronic conditions, multimorbidity, CHE and a reduced number of missed days of primary activity. This is consistent with other studies from LMICs. For example, an analysis by Zhong et al. (2020) reported that the incidence of CHE is consistently higher for former smokers compared to never smokers, accounting for 36.9% in 2011 and 46.8% in 2013 in China [51]. Our study also showed that the majority of former smokers had at least one NCD. Interestingly, our results showed negative associations between being current tobacco users and healthcare utilisation as well as productivity loss. One possible explanation for these findings is the “healthy smoker effect” hypothesis. This phenomenon has been observed in previous studies and refers to the attitude among current smokers in perceiving their condition as healthy to smoke, while those with symptoms of diseases might deny their disease and deter their journey to seek medical help [52–54]. Also, unhealthy smokers might have been affected by their smoking and hence were hospitalised or died and may not be present in the survey, leading to potential selection bias in the IFLS data. The fact that former tobacco users in this study were more likely to experience CHE also supports the possibility of the “healthy smoker effect” among current tobacco users who may have delayed their attempt to seek medical care and eventually ceased their habit when their disease had already impacted their life.

### Policy implications

The findings from this study can direct future NCD prevention policies. Our study suggested that tobacco consumption and physical inactivity contributed significantly towards direct and indirect costs. Therefore, actions aimed at smoking cessation and physical activity promotion need to be prioritised. Potential policy recommendations could include the Indonesian government signing and ratifying the Framework Convention on Tobacco Control. This framework mandate countries to take more stringent measures on tobacco control, particularly in monitoring, smoke-free policies, cessation program, warnings, advertising ban, and tobacco taxation [55]. The government should focus on promoting the coverage and accessibility of smoking cessation interventions, which are culturally appropriate [56]. While Indonesia has integrated “offer help to quit tobacco” as a part of the WHO Package of Essential NCD (WHO PEN) interventions in the primary health care, [57] only 34.6% of smokers who visited healthcare providers received advice to quit smoking [58]. Meanwhile, since national programs intended to promote physical activity, such as National Community Movement for Healthy Life (Gerakan Masyarakat Hidup Sehat – GERMAS) already exist [59], measures can be taken to optimise the program. Other evidence-based approaches could be adapted into GERMAS, such as offering a tax reduction for companies that comply with health promotion programs and implementing health risk appraisal for employees, including physical activity assessment and feedback [60]. Lastly, programs such as the integrated development post for NCD (Pos Binaan Terpadu/Posbindu), which is implemented at the community level to improve detection and prevention for NCDs risk factors, is promising to tackle the rising NCD burden in Indonesia. However, evaluations on the programs’ effectiveness are still lacking, and a recent local study reported that the participation rate is really low [61]. A bottom-up community participation approach could encouraged in order to promote the participation rate of the program [62].

Lastly, the Indonesian government needs to scale up its social protection and health insurance coverage policy for the older population who have insufficient health insurance coverage. The existing strategy for social protection for the older people in Indonesia is fragmented and consists of social assistance (non-contributory) and social insurance (contributory), covering programs such as food assistance, conditional cash transfers, and health insurance [63, 64]. While the Indonesian government has developed a five-year reform plan for its social protection system, some concerns still need to be addressed. The current investment in social protection accounts for 0.35% of the nation's GDP, while other countries such as Nepal have already invested 2% of their GDP in social protection [63]. Moreover, although studies revealed that Indonesian older people aged ≥ 65 years are more prone to poverty, the investment in social protection for older people is only minimal (0.001% of GDP). Under the current social protection program, the old age savings and older people pension scheme only insures those who are former formal workers, such as civil servants, military and police personnel, and company employees [63, 64]. This leaves out those in the “middle group” who are more vulnerable to financial insecurity. This “missing middle group” refers to a large part of the population (approximately 60%) of Indonesian who work in the informal sector but do not live in poverty; thus, they are not eligible to receive any government assistance through the current social protection scheme [63, 65].

### Strength and limitations

This is the first study in Indonesia that explores the relative impacts of the key behavioural risk factors for NCDs and compares these effects to being older. The use of the PSM approach also reduced selection bias and strengthened the internal validity of the study. Sensitivity analyses also suggested that the Kernel-based PSM approach was robust to other statistical methods, such as logistic regression and ZINB. However, the results of this study should be interpreted with caution due to several limitations. Firstly, the use of self-reported responses, in the measurement of physical activity, tobacco consumption, and the costs of seeking and utilising healthcare, may be prone to recall bias and might lead to under- or overreporting of the true prevalence in the population. Secondly, we calculated CHE by using the household expense and not accounting for specific individuals. In reality, some participants might belong to the same household, which might lead to bias in the results. Thirdly, the lack of temporality and the possibility of reverse causation is a limitation of a cross-sectional design. Therefore, no causation, and only association, can be inferred from our study findings. Fourth, our study population (≥ 40 years) may have lower productivity levels compared with the younger reproductive age group and have a lower baseline in terms of work days. This may have caused an underestimation of the level of productivity loss, even with the PSM methods that were used in the study. Finally, since IFLS did not conduct its survey in the Eastern part of Indonesia, the generalisability of the findings to the population in the Eastern part of Indonesia is limited. This particular region is commonly associated with lower development levels and limited health resources; thus, the current dataset may only portray regions in Indonesia that are more developed.

## Conclusion

In conclusion, being a former tobacco user, physically inactive, overweight, and obese were associated with a significantly higher health burden, health care costs and larger productivity loss in Indonesia. Therefore, prevention strategies for NCD risk factors are likely to have substantial health and economic benefits in Indonesia.

## Supplementary Information

**Additional file 1: Figure S1.** Flowchart of sampling selection for independent variable BMI. **Figure S2.** Flowchart of sampling selection for independent variable tobacco consumption. **Figure S3.** Flowchart of sampling selection for independent variable physical activity. **Figure S4.** Flowchart of sampling selection for independent variable ageing. **Table S1.** List of variables for 2014 IFLS analysis. **Table S2.** Sample characteristics stratified by age groups, before matching. **Table S3.** Sample characteristics stratified by tobacco consumption groups, before matching. **Table S4.** Sample characteristics stratified by BMI groups, before matching. **Table S5.** Sample characteristics stratified by physical activity (PA) groups, before matching. **Table S6.** Mean biases of covariates after matching using individual t-test (age group 50-59 vs 40-49). **Table S7.** Mean biases of covariates after matching using individual t-test (age group 60-69 vs 40-49). **Table S8.** Mean biases of covariates after matching using individual t-test (age group 70+ vs 40-49). **Table S9.** Mean biases of covariates after matching using individual t-test (former vs never use tobacco). **Table S10.** Mean biases of covariates after matching using individual t-test (light user vs never use tobacco). **Table S11.** Mean biases of covariates after matching using individual t-test (moderate user vs never use tobacco). **Table S12.** Mean biases of covariates after matching using individual t-test (heavy user vs never use tobacco). **Table S13.** Mean biases of covariates after matching using individual t-test (overweight vs normal BMI). **Table S14.** Mean biases of covariates after matching using individual t-test (obesity vs normal BMI). **Table S15.** Mean biases of covariates after matching using individual t-test (underweight vs normal BMI). **Table S16.** Mean biases of covariates after matching using individual t-test (low vs high physical activity. **Table S17.** Mean biases of covariates after matching using individual t-test (moderate vs high physical activity). **Table S18.** The ATT of the number of chronic conditions across different matching algorithms. **Table S19.** The ATT of multimorbidity presence across different matching algorithms. **Table S20.** The ATT of the number of outpatient visits across different matching algorithms. **Table S21.** The ATT of the number of inpatient visits across different matching algorithms. **Table S22.** The ATT of CHE >25% of total household expenditure across different matching algorithms. **Table S23.** The ATT of CHE >40% of total non-food expenditure across different matching algorithms. **Table S24.** The ATT of labour participation across different matching algorithms. **Table S25.** The ATT of the number of days primary activity missed across different matching algorithms. **Table S26.** Logistic and ZINB regression for the number of chronic condition and presence of multimorbidity. **Table S27.** Logistic and ZINB regression for the number of outpatient and inpatient visits. **Table S28.** Logistic regression for CHE >25% and 40%. **Table S29.** Logistic and ZINB regression for the productivity loss.

## Data Availability

The datasets generated during and/or analysed during the current study are available in the RAND Corporation repository, https://www.rand.org/well-being/social-and-behavioral-policy/data/FLS/IFLS/access.html [20].

## Abbreviations

CHE: Catastrophic health expenditure
NCDs: Non-communicable diseases
LMICs: Low-middle income countries
UHC: Universal health coverage
WHO: World Health Organization
IFLS: Indonesian Family Life Survey
BMI: Body mass index
IPAQ: International Physical Activity Questionnaire
PSM: Propensity score matching
NN: Nearest neighbour
ATT: Average treatment effect on the treated
ZINB: Zero-inflated negative binomial
AOR: Adjusted odds ratio
IRR: Incidence rate ratio
WHO PEN: WHO Package of Essential NCD
Gerakan Masyarakat Hidup Sehat – GERMAS: National Community Movement for Healthy Life
Pos Binaan Terpadu/Posbindu: Integrated development post for NCD
