# Supplementary material for: The relative impact of underweight, overweight, smoking, and physical inactivity on health and associated costs in Indonesia: propensity score matching of a national sample
Source: BMC Health Serv Res. 2022 Sep 17;22:1170. doi: 10.1186/s12913-022-08546-6 (PMC9482737; doi:10.1186/s12913-022-08546-6)
Supplement: Supplementary file 1 — Additional file 1: Figure S1. Flowchart of sampling selection for independent variable BMI. Figure S2. Flowchart of sampling selection for independent variable tobacco consumption. Figure S3. Flowchart of sampling selection for independent variable physical activity. Figure S4. Flowchart of sampling selection for independent variable ageing. Table S1. List of variables for 2014 IFLS analysis. Table S2. Sample characteristics stratified by age groups, before matching. Table S3. Sample characteristics stratified by tobacco consumption groups, before matching. Table S4. Sample characteristics stratified by BMI groups, before matching. Table S5. Sample characteristics stratified by physical activity (PA) groups, before matching. Table S6. Mean biases of covariates after matching using individual t-test (age group 50-59 vs 40-49). Table S7. Mean biases of covariates after matching using individual t-test (age group 60-69 vs 40-49). Table S8. Mean biases of covariates after matching using individual t-test (age group 70+ vs 40-49). Table S9. Mean biases of covariates after matching using individual t-test (former vs never use tobacco). Table S10. Mean biases of covariates after matching using individual t-test (light user vs never use tobacco). Table S11. Mean biases of covariates after matching using individual t-test (moderate user vs never use tobacco). Table S12. Mean biases of covariates after matching using individual t-test (heavy user vs never use tobacco). Table S13. Mean biases of covariates after matching using individual t-test (overweight vs normal BMI). Table S14. Mean biases of covariates after matching using individual t-test (obesity vs normal BMI). Table S15. Mean biases of covariates after matching using individual t-test (underweight vs normal BMI). Table S16. Mean biases of covariates after matching using individual t-test (low vs high physical activity. Table S17. Mean biases of covariates after matching using individual t-test (moderate vs high phys [file 12913_2022_8546_MOESM1_ESM.docx]

#### Figure S1. Flowchart of sampling selection for independent variable BMI

**
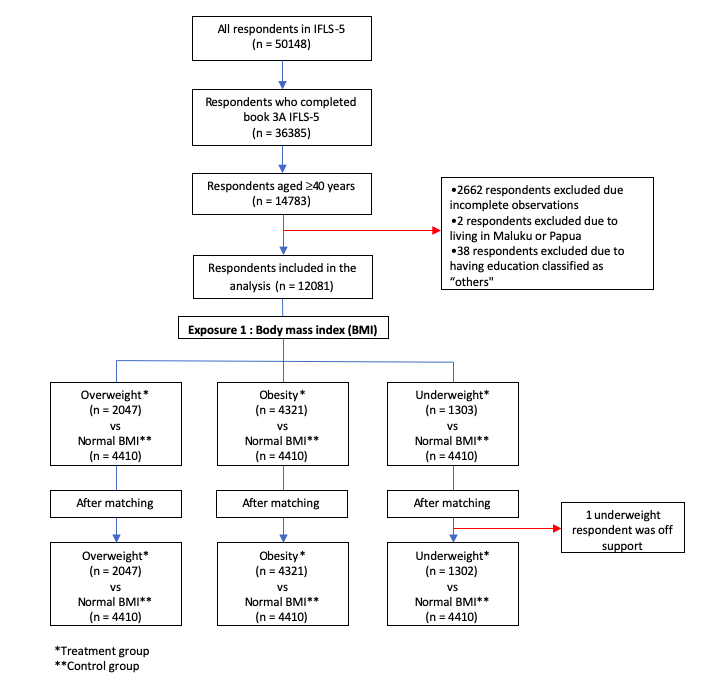
**

#### Figure S2. Flowchart of sampling selection for independent variable tobacco consumption

**
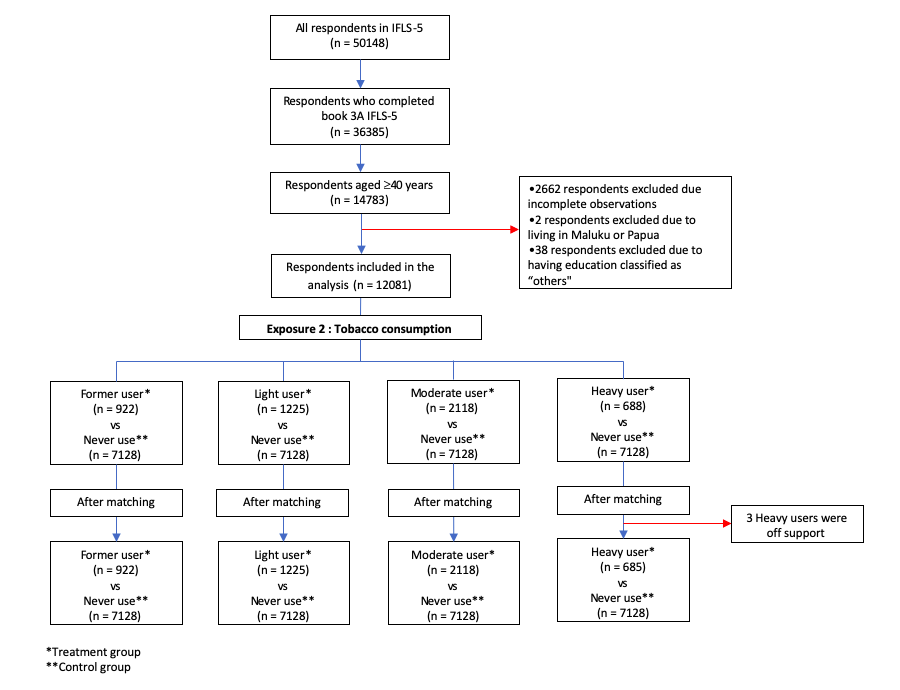
**

#### Figure S3. Flowchart of sampling selection for independent variable physical activity

**
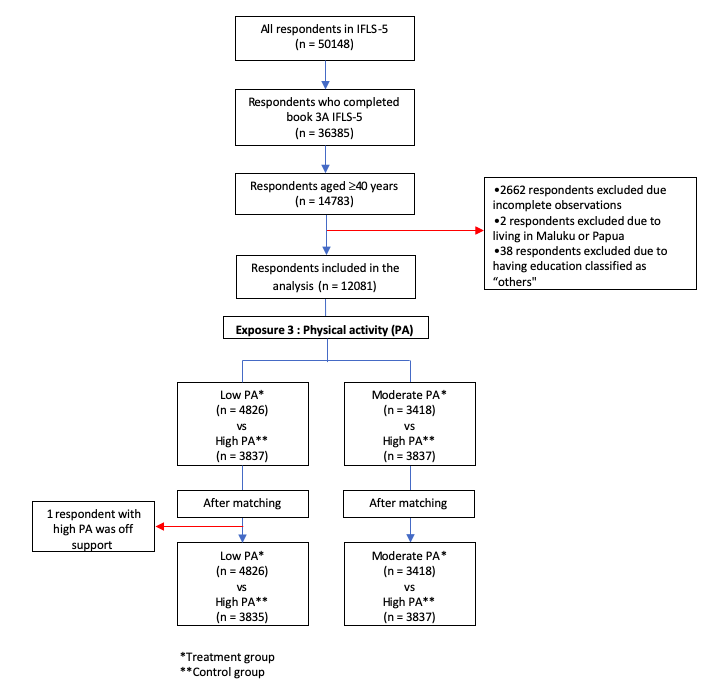
**

#### Figure S4. Flowchart of sampling selection for independent variable ageing

**
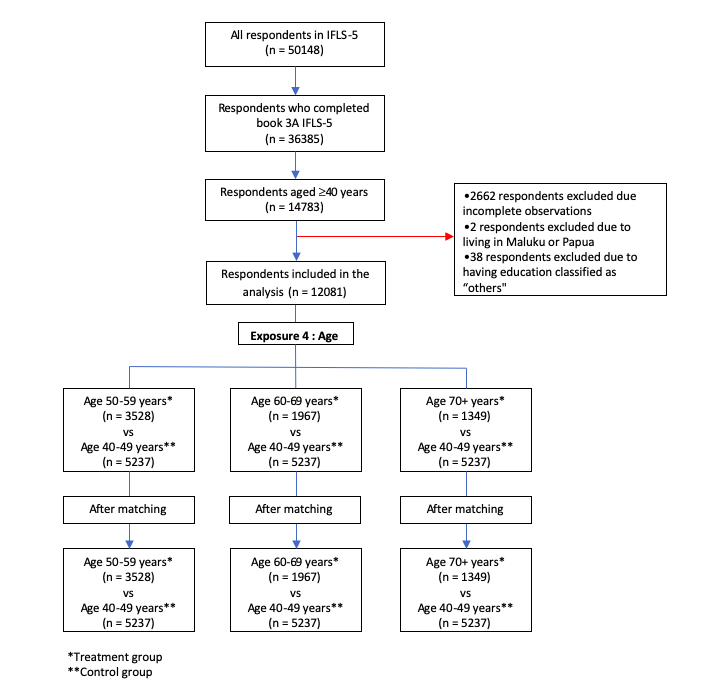
**

####

#### Table S1. List of variables for 2014 IFLS analysis

| **Variables** | **Type** | **Measurement** | **Source of Measurement** |
| --- | --- | --- | --- |
| **Dependant variables** | | | |
| **1. Health status** | | | |
| - Productivity loss |  |  |  |
| - Labor participation | Binary | 1. No 2. Yes | TK06a: Did you work/try to work/help to earn income for pay for at least 1 hour during the past week? |
| - Activity missed due to poor health | Numerical | Number of days | KK02a: During the last 4 weeks, how many days of  your primary daily activities did you miss due to  poor health? |
| - Stayed in bed | Numerical | Number of days | In the last 4 weeks, how many days have you stayed in bed due to poor health? |
| **2. Health service use** | | | |
| - Outpatient care | Binary | 1. No 2. Yes | Book IIIB:  RJ00: In the last 4 weeks have you visited a public hospital, puskesmas, private hospital, clinic, health worker or doctor’s practice or been visited by a health worker or doctor? |
|  | Numerical | Number of days | Book IIIB:  RJ02: How many times did you visit / been visited by [...] during the last 4 weeks? |
| - Inpatient care | Binary | 1. No 2. Yes | Book IIIB:  RN00: During the past 12 months have you ever received patient care at a hospital, puskesmas, clinic, or other? |
|  | Numerical | Number of days | Book IIIB:  RN02: How many times have you received inpatient care at […] during the past 12 months? |
| **3. Financial burden** | | | |
| - OOPE of outpatient care | Numerical | International dollars | Book IIIB:  RJ02b: How much did you pay out of pocket for outpatient care at […] during the past 4 weeks? |
| - OOPE of inpatient care | Numerical | International dollars | Book IIIB:  RN02b: How much did you pay out of pocket for inpatient care at […] during the past 12 months? |
| - Total OOPE | Numerical | International dollars | Annual total OOPE for outpatient and inpatient visits |
| - Catastrophic health expenditure | Binary | 1. No 2. Yes | Book KS:  "How much money spent by all household members for medical costs during the past year?" |
| **Main independent variables** | | | |
| 1. Age | Categorical ordinal | 0. 40-49  1. 50-59  2. 60-69  3. 70+ | Book IIIA:  Age: How old are you? |
| 2. Tobacco consumption | Categorical ordinal | 1. Never use 2. Former user 3. Light user 4. Moderate user 5. Heavy user | Book IIIb:  KM01a. Have you ever chewed tobacco, smoked a pipe, smoked self-rolled cigarettes, or smoked cigarettes/cigars?  KM04. Do you still have the habit or have you totally quit? |
| 3. Physical inactivity | Binary | 1. High 2. Moderate 3. High | Book IIIb:  KK02mA – KK02mC. During the last 7 days, did you do any […] for at least 10 minutes continuously?  KK02nA – KK02nC. How much time did you usually spend doing […] on one of those days?  KK02oA – KK02oC. During the last 7 days, on how many days did you do […]? |
| 4. Overweight or obese | Categorical ordinal | 1. Normal BMI (18.5-23.0) 2. Overweight (23.0-<25.0) 3. Obese (≥25) 4. Underweight (<18.5) | Book US:  US06. Weight (kg)  US04. Height (cm) |
| 5. Number of NCDs | Categorical ordinal | 1. 0 2. 1 3. 2 4. 3+ | Book IIIB:  CD05a – CD05r: Have a  doctor/paramedic/nurse/ midwife ever told you that you had [list of chronic diseases] |
| List of NCDs includes 14 diseases, i.e. hypertension, diabetes, asthma, heart attack/coronary heart diseases, liver disease, stroke, cancer, arthritis/rheumatism, high cholesterol, prostate illness (for male respondents), kidney diseases (excluding malignancy), digestive diseases, mental illness, and memory-related diseases. | | | |
| **Covariates** | | | |
| Sex | Binary | 1. Female 2. Male | Book IIIA:  Sex: (identified by interviewers) |
| Ethnicity | Categorical nominal | 1. Javanese 2. Sundanese 3. Others |  |
| Marital status | Binary | 1. Unmarried/Divorce 2. Married or living together | Book IIIA  HR00b: Are you currently married? |
| Education | Categorical ordinal | 1. None 2. Elementary school 3. Junior high school 4. High school 5. Tertiary | Book IIIA:  DL06: What is the highest education level  attended?  DL07: What is the highest grade completed at school. |
| Occupation | Categorical nominal | 1. None 2. Casual worker 3. Self-employed 4. Government/private worker | Book IIIA:  TK06a: Did you work/try to work/help to  earn income for pay for at least  1 hour during the past week?  TK15: Which category best describes the work you did in your last job? |
| Health insurance status | Binary | 1. Uninsured (Not covered by any insurance) 2. Insured | Book IIIB:  AK01: Are you the policy holder/primary beneficiary of health benefits, health insurance? |
| Household per capita expenditure | Categorical ordinal | 1. Q1 (lowest) 2. Q2 3. Q3 4. Q4 5. Q5 (highest) | Book KS |
| Residency | Binary | 1. Rural 2. Urban | Book T-2: SC06: (identified by interviewers) |
| Region of residency | Categorical nominal | 1. Java and Bali 2. Sumatra 3. Nusa Tenggara 4. Kalimantan 5. Sulawesi | Book T-2:  SC01: province (identified by interviewers) |

#### Table S2. Sample characteristics stratified by age groups, before matching

| **Variable** | **Age 40-49 years (n=5237)** | | **Age 50-59 years (n=3528)** | | **Age 60-69 years (n=1967)** | | **Age 70+ years (n=1349)** | |
| --- | --- | --- | --- | --- | --- | --- | --- | --- |
|  | **%** | **n** | **%** | **n** | **%** | **n** | **%** | **n** |
| **Sex** (%) |  |  |  |  |  |  |  |  |
| Female | 50.3 | 2635 | 54.3 | 1915 | 52.5 | 1033 | 54.9 | 740 |
| Male | 49.7 | 2602 | 45.7 | 1613 | 47.5 | 934 | 45.1 | 609 |
| **Marital status (%)** |  |  |  |  |  |  |  |  |
| Not currently married | 9.3 | 489 | 18.4 | 651 | 31.8 | 625 | 51.2 | 690 |
| Currently Married | 90.7 | 4748 | 81.6 | 2877 | 68.2 | 1342 | 48.8 | 659 |
| **Education (%)** |  |  |  |  |  |  |  |  |
| No education | 21.4 | 1120 | 47.8 | 1686 | 50.9 | 1001 | 69.7 | 940 |
| Primary | 24.9 | 1307 | 23.8 | 840 | 25.7 | 506 | 19.6 | 264 |
| Junior high school | 15.9 | 832 | 9.6 | 338 | 8.5 | 167 | 3.7 | 50 |
| Senior high school | 28.9 | 1513 | 11.9 | 421 | 10.5 | 207 | 4.7 | 64 |
| Tertiary | 8.9 | 465 | 6.9 | 243 | 4.4 | 86 | 2.3 | 31 |
| **Ethnicity** |  |  |  |  |  |  |  |  |
| Javanese | 46.2 | 2421 | 46.4 | 1636 | 47.3 | 930 | 50.1 | 676 |
| Sundanese | 12.8 | 667 | 11.8 | 415 | 12.2 | 241 | 11.3 | 152 |
| Others | 41 | 2149 | 41.8 | 1477 | 40.5 | 796 | 38.6 | 521 |
| **Had any health insurance** |  |  |  |  |  |  |  |  |
| No | 47.3 | 2478 | 51.6 | 1822 | 52 | 1023 | 54.3 | 732 |
| Yes | 52.7 | 2759 | 48.4 | 1706 | 48 | 944 | 45.7 | 617 |
| **Type of work** |  |  |  |  |  |  |  |  |
| Unemployed | 14.6 | 764 | 17.9 | 632 | 34.6 | 681 | 56.9 | 768 |
| Casual | 18.4 | 965 | 18.1 | 637 | 13.8 | 271 | 9.7 | 131 |
| Self-employed | 36.8 | 1925 | 43 | 1517 | 43.7 | 860 | 31 | 418 |
| Government/private | 30.2 | 1583 | 21 | 742 | 7.9 | 155 | 2.4 | 32 |
| **Percapita consumption expenditure** | | | | |  |  |  |  |
| Q1 (the lowest) | 17.6 | 920 | 18.6 | 657 | 21.4 | 422 | 30.2 | 407 |
| Q2 | 20 | 1050 | 19.2 | 679 | 19.6 | 386 | 23 | 310 |
| Q3 | 21.2 | 1108 | 19 | 672 | 20.6 | 405 | 18.4 | 248 |
| Q4 | 21.3 | 1114 | 20.8 | 732 | 19 | 372 | 14.2 | 192 |
| Q5 (the highest) | 19.9 | 1045 | 22.4 | 788 | 19.4 | 382 | 14.2 | 192 |
| **Residency** |  |  |  |  |  |  |  |  |
| Rural | 40.4 | 2115 | 43.9 | 1550 | 44 | 865 | 51.7 | 698 |
| Urban | 59.6 | 3122 | 56.1 | 1978 | 56 | 1102 | 48.3 | 651 |
| **Island** |  |  |  |  |  |  |  |  |
| Java-Bali | 61.7 | 3233 | 62.3 | 2198 | 64.8 | 1275 | 65.2 | 880 |
| Sumatra | 22.3 | 1167 | 21.7 | 767 | 20.2 | 398 | 18.1 | 244 |
| Nusa Tenggara | 6.6 | 348 | 6.4 | 225 | 6 | 119 | 8.1 | 110 |
| Kalimantan | 4.8 | 250 | 4.8 | 169 | 4 | 78 | 4 | 53 |
| Sulawesi | 4.6 | 239 | 4.8 | 169 | 5 | 97 | 4.6 | 62 |
| **Tobacco consumption** |  |  |  |  |  |  |  |  |
| Never use tobacco | 60.1 | 3147 | 60.2 | 2123 | 58 | 1140 | 53.2 | 718 |
| Former user | 4.5 | 237 | 7 | 249 | 10.8 | 212 | 16.6 | 224 |
| Light user | 11 | 578 | 9.7 | 342 | 10 | 197 | 8 | 108 |
| Moderate user | 18 | 942 | 17.9 | 631 | 16 | 314 | 17.1 | 231 |
| Heavy user | 6.4 | 333 | 5.2 | 183 | 5.2 | 104 | 5.1 | 68 |
| **BMI (kg/m2)** |  |  |  |  |  |  |  |  |
| Underweight (<18.5) | 5.9 | 311 | 8.7 | 305 | 14.6 | 287 | 29.6 | 400 |
| Normal (18.5-23.0) | 33.5 | 1754 | 35.7 | 1260 | 39.2 | 772 | 46.3 | 624 |
| Overweight (23.0 - <25.0) | 18.3 | 957 | 17 | 602 | 17.1 | 336 | 11.3 | 152 |
| Obesity (>= 25) | 42.3 | 2215 | 38.6 | 1361 | 29.1 | 572 | 12.8 | 173 |
| **Physical inactivity** |  |  |  |  |  |  |  |  |
| High | 34.7 | 1820 | 34.4 | 1214 | 30.2 | 593 | 15.6 | 210 |
| Moderate | 34.9 | 1827 | 36 | 1269 | 41.5 | 817 | 67.7 | 913 |
| Low | 30.4 | 1590 | 29.6 | 1045 | 28.3 | 557 | 16.7 | 226 |
| **Number of NCDs** |  |  |  |  |  |  |  |  |
| 0 | 48.8 | 2558 | 34.5 | 1217 | 26 | 512 | 18.7 | 252 |
| 1 | 36.3 | 1899 | 41.7 | 1470 | 44.2 | 869 | 53 | 715 |
| 2 | 10.6 | 556 | 15.7 | 555 | 18.9 | 372 | 19.3 | 261 |
| 3+ | 4.3 | 224 | 8.1 | 286 | 10.9 | 214 | 9 | 121 |

#### Table S3. Sample characteristics stratified by tobacco consumption groups, before matching

| **Variable** | **Never use (n=7128)** | | **Former user (n=922)** | | **Light user (n=1225)** | | **Moderate user (n=2118)** | | **Heavy user (n=688)** | |
| --- | --- | --- | --- | --- | --- | --- | --- | --- | --- | --- |
|  | **%** | **n** | **%** | **n** | **%** | **n** | **%** | **n** | **%** | **n** |
| **Age (%)** |  |  |  |  |  |  |  |  |  |  |
| 40 – 49 years | 44.1 | 3147 | 25.7 | 237 | 47.2 | 578 | 44.5 | 942 | 48.4 | 333 |
| 50 – 59 years | 29.8 | 2123 | 27 | 249 | 27.9 | 342 | 29.8 | 631 | 26.6 | 183 |
| 60 – 69 years | 16 | 1140 | 23 | 212 | 16.1 | 197 | 14.8 | 314 | 15.1 | 104 |
| 70+ years | 10.1 | 718 | 24.3 | 224 | 8.8 | 108 | 10.9 | 231 | 9.9 | 68 |
| **Sex (%)** |  |  |  |  |  |  |  |  |  |  |
| Female | 82.7 | 5896 | 11.9 | 110 | 10.2 | 125 | 7.6 | 162 | 4.4 | 30 |
| Male | 17.3 | 1232 | 88.1 | 812 | 89.8 | 1100 | 92.4 | 1956 | 95.6 | 658 |
| **Marital status (%)** |  |  |  |  |  |  |  |  |  |  |
| Not currently married | 25.2 | 1798 | 16.7 | 154 | 12.4 | 152 | 12.5 | 265 | 12.5 | 86 |
| Currently Married | 74.8 | 5330 | 83.3 | 768 | 87.6 | 1073 | 87.5 | 1853 | 87.5 | 602 |
| **Education (%)** |  |  |  |  |  |  |  |  |  |  |
| No education | 41 | 2921 | 34.5 | 318 | 32.2 | 394 | 41.1 | 869 | 35.6 | 245 |
| Primary | 23.4 | 1667 | 24.6 | 227 | 25.1 | 307 | 26.1 | 553 | 23.7 | 163 |
| Junior high school | 11.2 | 802 | 11.5 | 106 | 12.7 | 156 | 10.8 | 229 | 13.7 | 94 |
| Senior high school | 16.7 | 1192 | 19.7 | 182 | 24.1 | 295 | 18.1 | 384 | 22.1 | 152 |
| Tertiary | 7.7 | 546 | 9.7 | 89 | 5.9 | 73 | 3.9 | 83 | 4.9 | 34 |
| **Ethnicity** |  |  |  |  |  |  |  |  |  |  |
| Javanese | 48 | 3425 | 48.7 | 449 | 42.3 | 518 | 47.9 | 1014 | 37.3 | 257 |
| Sundanese | 11.8 | 838 | 9.9 | 91 | 18.2 | 223 | 12 | 255 | 9.9 | 68 |
| Others | 40.2 | 2865 | 41.4 | 382 | 39.5 | 484 | 40.1 | 849 | 52.8 | 363 |
| **Had any health insurance** | |  |  |  |  |  |  |  |  |  |
| No | 49.4 | 3520 | 46.3 | 427 | 48.2 | 590 | 54 | 1144 | 54.4 | 374 |
| Yes | 50.6 | 3608 | 53.7 | 495 | 51.8 | 635 | 46 | 974 | 45.6 | 314 |
| **Type of work** |  |  |  |  |  |  |  |  |  |  |
| Unemployed | 30.2 | 2152 | 30.3 | 279 | 13.6 | 167 | 8.7 | 185 | 9 | 62 |
| Casual | 17.8 | 1270 | 10.2 | 94 | 16.7 | 205 | 16.6 | 351 | 12.2 | 84 |
| Self-employed | 33.7 | 2403 | 39 | 360 | 43.1 | 528 | 50 | 1059 | 53.8 | 370 |
| Government/private | 18.3 | 1303 | 20.5 | 189 | 26.6 | 325 | 24.7 | 523 | 25 | 172 |
| **Percapita consumption expenditure** | | |  |  |  |  |  |  |  |  |
| Q1 (the lowest) | 19.5 | 1389 | 20.9 | 193 | 18.9 | 232 | 22.1 | 469 | 17.9 | 123 |
| Q2 | 19.4 | 1385 | 17.7 | 163 | 22.9 | 280 | 22.2 | 470 | 18.5 | 127 |
| Q3 | 20.2 | 1439 | 17.2 | 159 | 20.6 | 252 | 20.7 | 438 | 21 | 145 |
| Q4 | 20.4 | 1453 | 21.3 | 196 | 19 | 233 | 18.9 | 401 | 18.5 | 127 |
| Q5 (the highest) | 20.5 | 1462 | 22.9 | 211 | 18.6 | 228 | 16.1 | 340 | 24.1 | 166 |
| **Residency** |  |  |  |  |  |  |  |  |  |  |
| Rural | 40.9 | 2913 | 39.6 | 365 | 40.1 | 491 | 52.9 | 1121 | 49.1 | 338 |
| Urban | 59.1 | 4215 | 60.4 | 557 | 59.9 | 734 | 47.1 | 997 | 50.9 | 350 |
| **Island** |  |  |  |  |  |  |  |  |  |  |
| Java-Bali | 64.8 | 4623 | 58.7 | 541 | 64.7 | 792 | 60.3 | 1277 | 51.3 | 353 |
| Sumatra | 18.8 | 1338 | 26.8 | 247 | 21.6 | 265 | 24.8 | 526 | 29.1 | 200 |
| Nusa Tenggara | 6.7 | 477 | 4.1 | 38 | 5.9 | 72 | 6.7 | 143 | 10.5 | 72 |
| Kalimantan | 4.7 | 337 | 5.2 | 48 | 3.6 | 44 | 4.1 | 86 | 5.1 | 35 |
| Sulawesi | 5 | 353 | 5.2 | 48 | 4.2 | 52 | 4.1 | 86 | 4 | 28 |
| **BMI (kg/m2)** |  |  |  |  |  |  |  |  |  |  |
| Underweight (<18.5) | 8.8 | 628 | 14.3 | 132 | 13.5 | 165 | 13.9 | 294 | 12.2 | 84 |
| Normal (18.5-23.0) | 29.5 | 2100 | 37.1 | 342 | 45.5 | 557 | 50.8 | 1076 | 48.7 | 335 |
| Overweight (23.0 - <25.0) | 17.4 | 1239 | 17.7 | 163 | 14.8 | 182 | 16.5 | 349 | 16.6 | 114 |
| Obesity (>= 25) | 44.3 | 3161 | 30.9 | 285 | 26.2 | 321 | 18.8 | 399 | 22.5 | 155 |
| **Physical inactivity** |  |  |  |  |  |  |  |  |  |  |
| High | 27.6 | 1966 | 17.9 | 257 | 37.6 | 461 | 42 | 890 | 38.2 | 263 |
| Moderate | 41.2 | 2937 | 45 | 415 | 38.4 | 470 | 34.9 | 739 | 38.5 | 265 |
| Low | 31.2 | 2225 | 27.1 | 250 | 24 | 294 | 23.1 | 489 | 23.3 | 160 |
| **Number of NCDs** |  |  |  |  |  |  |  |  |  |  |
| 0 | 33.9 | 2418 | 25.7 | 237 | 44.7 | 547 | 48 | 1016 | 46.7 | 321 |
| 1 | 41.9 | 2985 | 40.5 | 374 | 39.4 | 483 | 39.6 | 838 | 39.7 | 273 |
| 2 | 16.3 | 1163 | 20 | 184 | 11 | 135 | 9.3 | 197 | 9.4 | 65 |
| 3+ | 7.9 | 562 | 13.8 | 127 | 4.9 | 60 | 3.1 | 67 | 4.2 | 29 |

#### Table S4. Sample characteristics stratified by BMI groups, before matching

| **Variable** | **Normal BMI (n=4410)** | | **Overweight (n=2047)** | | **Obese (n=4321)** | | **Underweight (n=1303)** | |
| --- | --- | --- | --- | --- | --- | --- | --- | --- |
|  | % | n | % | n | % | n | % | n |
| **Age (%)** |  |  |  |  |  |  |  |  |
| 40 – 49 years | 39.8 | 1754 | 46.8 | 957 | 51.3 | 2215 | 23.9 | 311 |
| 50 – 59 years | 28.6 | 1260 | 29.4 | 602 | 31.5 | 1361 | 23.4 | 305 |
| 60 – 69 years | 17.5 | 772 | 16.4 | 336 | 13.2 | 572 | 22 | 287 |
| 70+ years | 14.1 | 624 | 7.4 | 152 | 4 | 173 | 30.7 | 400 |
| **Sex (%)** |  |  |  |  |  |  |  |  |
| Female | 41.4 | 1825 | 51.3 | 1051 | 65.6 | 2834 | 47 | 613 |
| Male | 58.6 | 2585 | 48.7 | 996 | 34.4 | 1487 | 53 | 690 |
| **Marital status (%)** |  |  |  |  |  |  |  |  |
| Not currently married | 20.8 | 918 | 18.5 | 378 | 16.6 | 717 | 33.9 | 442 |
| Currently Married | 79.2 | 3492 | 81.5 | 1669 | 83.4 | 3604 | 66.1 | 861 |
| **Education (%)** |  |  |  |  |  |  |  |  |
| No education | 43.7 | 1928 | 35 | 716 | 30.7 | 1327 | 59.5 | 776 |
| Primary | 25.3 | 1117 | 23.6 | 483 | 13.4 | 1012 | 23.4 | 305 |
| Junior high school | 10.9 | 481 | 12.2 | 250 | 13.3 | 572 | 6.5 | 84 |
| Senior high school | 15.8 | 697 | 20.5 | 419 | 22.6 | 978 | 8.5 | 111 |
| Tertiary | 4.3 | 187 | 8.7 | 179 | 10 | 432 | 2.1 | 27 |
| **Ethnicity** |  |  |  |  |  |  |  |  |
| Javanese | 47.9 | 2112 | 48.1 | 984 | 46.8 | 2022 | 41.8 | 545 |
| Sundanese | 12 | 529 | 11.5 | 236 | 13.2 | 571 | 10.7 | 139 |
| Others | 40.1 | 1769 | 40.4 | 827 | 40 | 1728 | 47.5 | 619 |
| **Had any health insurance** |  |  |  |  |  |  |  |  |
| No | 52 | 2295 | 49.8 | 1020 | 47.3 | 2045 | 53.3 | 695 |
| Yes | 48 | 2115 | 50.2 | 1027 | 52.7 | 2276 | 46.7 | 608 |
| **Type of work** |  |  |  |  |  |  |  |  |
| Unemployed | 20.7 | 914 | 21 | 430 | 24.9 | 1076 | 32.6 | 425 |
| Casual | 17.9 | 790 | 16.5 | 338 | 14.4 | 621 | 19.6 | 255 |
| Self-employed | 42.5 | 1872 | 39.3 | 804 | 36.4 | 1574 | 36.1 | 470 |
| Government/private | 18.9 | 834 | 23.2 | 475 | 24.3 | 1050 | 11.7 | 153 |
| **Percapita consumption expenditure** |  |  |  |  |  |  |  |  |
| Q1 (the lowest) | 24 | 1054 | 16.7 | 341 | 14.1 | 609 | 30.9 | 402 |
| Q2 | 21.8 | 962 | 19.8 | 406 | 16.4 | 708 | 26.8 | 349 |
| Q3 | 20 | 886 | 21.1 | 431 | 20.3 | 876 | 18.4 | 240 |
| Q4 | 18.5 | 816 | 21.1 | 432 | 22.6 | 978 | 14.1 | 184 |
| Q5 (the highest) | 15.7 | 692 | 21.3 | 437 | 26.6 | 1150 | 9.8 | 128 |
| **Residency** |  |  |  |  |  |  |  |  |
| Rural | 49.2 | 2169 | 41.1 | 842 | 33.8 | 1462 | 57.9 | 755 |
| Urban | 50.8 | 2241 | 58.9 | 1205 | 66.2 | 2859 | 42.1 | 548 |
| **Island** |  |  |  |  |  |  |  |  |
| Java-Bali | 62.6 | 2763 | 62 | 1269 | 64.3 | 2780 | 59.4 | 774 |
| Sumatra | 20.9 | 921 | 22.5 | 461 | 22.1 | 955 | 18.4 | 239 |
| Nusa Tenggara | 7.1 | 312 | 5.9 | 120 | 5.3 | 227 | 11 | 143 |
| Kalimantan | 4.1 | 180 | 5 | 103 | 4.3 | 187 | 6.1 | 80 |
| Sulawesi | 5.3 | 234 | 4.6 | 94 | 4 | 172 | 5.1 | 67 |
| **Tobacco consumption** |  |  |  |  |  |  |  |  |
| Never use tobacco | 47.6 | 2100 | 60.5 | 1239 | 73.2 | 3161 | 48.2 | 628 |
| Former user | 7.8 | 342 | 8 | 163 | 6.6 | 285 | 10.1 | 132 |
| Light user | 12.6 | 557 | 8.9 | 182 | 7.4 | 321 | 12.7 | 165 |
| Moderate user | 24.4 | 1076 | 17 | 349 | 9.2 | 399 | 22.6 | 294 |
| Heavy user | 7.6 | 335 | 5.6 | 114 | 3.6 | 155 | 6.4 | 84 |
| **Physical inactivity** |  |  |  |  |  |  |  |  |
| High | 36.6 | 1616 | 31.1 | 636 | 27.5 | 1190 | 30.3 | 395 |
| Moderate | 25.1 | 1105 | 29.7 | 803 | 39.6 | 1710 | 47.9 | 624 |
| Low | 38.3 | 1689 | 39.2 | 608 | 32.9 | 1421 | 21.8 | 284 |
| **Number of NCDs** |  |  |  |  |  |  |  |  |
| 0 | 44.3 | 1954 | 36.1 | 738 | 29.5 | 1273 | 44 | 574 |
| 1 | 39.2 | 1729 | 42.7 | 875 | 42.7 | 1844 | 38.8 | 505 |
| 2 | 11.5 | 507 | 14.6 | 299 | 17.5 | 757 | 13.9 | 181 |
| 3+ | 5 | 220 | 6.6 | 135 | 10.3 | 447 | 3.3 | 43 |

#### Table S5. Sample characteristics stratified by physical activity (PA) groups, before matching

| **Variable** | **High PA (n=3837)** | | **Low PA (n=4826)** | | **Moderate PA (n=3418)** | |
| --- | --- | --- | --- | --- | --- | --- |
|  | % | n | % | n | % | n |
| **Age (%)** |  |  |  |  |  |  |
| 40 – 49 years | 47.4 | 1820 | 37.9 | 1827 | 46.5 | 1590 |
| 50 – 59 years | 31.6 | 1214 | 26.3 | 1269 | 30.6 | 1045 |
| 60 – 69 years | 15.5 | 593 | 16.9 | 817 | 16.3 | 557 |
| 70+ years | 5.5 | 210 | 18.9 | 913 | 6.6 | 226 |
| **Sex (%)** |  |  |  |  |  |  |
| Female | 43.3 | 1661 | 55.2 | 2666 | 58.4 | 1996 |
| Male | 56.7 | 2176 | 44.8 | 2160 | 41.6 | 1422 |
| **Marital status (%)** |  |  |  |  |  |  |
| Not currently married | 14.4 | 551 | 26.3 | 1270 | 18.5 | 634 |
| Currently Married | 85.6 | 3286 | 73.7 | 3556 | 81.5 | 2784 |
| **Education (%)** |  |  |  |  |  |  |
| No education | 40.4 | 1550 | 42.1 | 2031 | 34.1 | 1166 |
| Primary | 26.7 | 1024 | 21.5 | 1040 | 25 | 853 |
| Junior high school | 11.4 | 436 | 11.1 | 535 | 12.2 | 416 |
| Senior high school | 16.4 | 631 | 18.4 | 886 | 20.1 | 688 |
| Tertiary | 5.1 | 196 | 6.9 | 334 | 8.6 | 295 |
| **Ethnicity** |  |  |  |  |  |  |
| Javanese | 50.9 | 1953 | 45.3 | 2187 | 44.6 | 1523 |
| Sundanese | 11.1 | 426 | 11.7 | 563 | 14.2 | 486 |
| Others | 38 | 1458 | 43 | 2076 | 41.2 | 1409 |
| **Had any health insurance** |  |  |  |  |  |  |
| No | 51.1 | 1959 | 49.1 | 2370 | 50.5 | 1726 |
| Yes | 48.9 | 1878 | 50.9 | 2456 | 49.5 | 1692 |
| **Type of work** |  |  |  |  |  |  |
| Unemployed | 11.5 | 443 | 33.3 | 1608 | 23.2 | 794 |
| Casual | 20.6 | 790 | 14 | 675 | 15.8 | 539 |
| Self-employed | 45.9 | 1761 | 33.3 | 1607 | 39.6 | 1352 |
| Government/private | 22 | 843 | 19.4 | 936 | 21.4 | 733 |
| **Percapita consumption expenditure** |  |  |  |  |  |  |
| Q1 (the lowest) | 21.3 | 816 | 20.5 | 989 | 17.6 | 601 |
| Q2 | 21.3 | 819 | 20.2 | 973 | 18.5 | 633 |
| Q3 | 21.2 | 812 | 18.7 | 903 | 21 | 718 |
| Q4 | 19.8 | 761 | 19.8 | 956 | 20.3 | 693 |
| Q5 (the highest) | 16.4 | 629 | 10.8 | 1005 | 22.6 | 773 |
| **Residency** |  |  |  |  |  |  |
| Rural | 49.6 | 1902 | 40.9 | 1972 | 39.6 | 1354 |
| Urban | 50.4 | 1935 | 59.1 | 2854 | 60.4 | 2064 |
| **Island** |  |  |  |  |  |  |
| Java-Bali | 66.2 | 2540 | 59.4 | 2868 | 63.7 | 2178 |
| Sumatra | 19.8 | 761 | 24.4 | 1177 | 18.7 | 638 |
| Nusa Tenggara | 7.7 | 297 | 5.1 | 245 | 7.6 | 260 |
| Kalimantan | 4.1 | 156 | 3.8 | 185 | 6.1 | 209 |
| Sulawesi | 2.2 | 83 | 7.3 | 351 | 3.9 | 133 |
| **Tobacco consumption** |  |  |  |  |  |  |
| Never use tobacco | 51.2 | 1966 | 60.9 | 2937 | 65.1 | 2225 |
| Former user | 6.7 | 257 | 8.6 | 415 | 7.3 | 250 |
| Light user | 12 | 461 | 9.7 | 470 | 8.6 | 294 |
| Moderate user | 23.2 | 890 | 15.3 | 739 | 14.3 | 489 |
| Heavy user | 6.9 | 263 | 5.5 | 265 | 4.7 | 160 |
| **BMI (kg/m2)** |  |  |  |  |  |  |
| Underweight (<18.5) | 10.3 | 395 | 12.9 | 624 | 8.3 | 284 |
| Normal (18.5-23.0) | 42.1 | 1616 | 35 | 1689 | 32.3 | 1105 |
| Overweight (23.0 - <25.0) | 16.6 | 636 | 16.7 | 803 | 17.8 | 608 |
| Obesity (>= 25) | 31 | 1190 | 35.4 | 1710 | 41.6 | 1421 |
| **Number of NCDs** |  |  |  |  |  |  |
| 0 | 42.4 | 1628 | 34.8 | 1679 | 36 | 1232 |
| 1 | 40.4 | 1550 | 41.8 | 2015 | 40.6 | 1388 |
| 2 | 11.9 | 457 | 15.9 | 769 | 15.2 | 518 |
| 3+ | 5.3 | 202 | 7.5 | 363 | 8.2 | 280 |

#### Table S6. Mean biases of covariates after matching using individual t-test (age group 50-59 vs 40-49)

| **Variable** | **Matching Algorithm** | | |
| --- | --- | --- | --- |
|  | Nearest neighbour with replacement | Nearest neighbour without replacement | Kernel |
| **Sex (%)** |  |  |  |
| Female |  |  |  |
| Male | 0.6 | 1.03 | -1.09 |
| **Marital status (%)** |  |  |  |
| Not currently married |  |  |  |
| Currently Married | -2.16** | 0.78 | -1.27 |
| **Education (%)** |  |  |  |
| No education |  |  |  |
| Primary | -0.53 | -0.88 | -0.54 |
| Junior high school | 0.7 | 0.25 | 0.07 |
| Senior high school | 0.29 | 0.3 | 0.43 |
| Tertiary | -0.28 | 0.29 | -0.58 |
| **Ethnicity** |  |  |  |
| Javanese |  |  |  |
| Sundanese | 0.15 | -0.28 | -0.24 |
| Others | 0.02 | -0.3 | -0.06 |
| **Had any health insurance** |  |  |  |
| No |  |  |  |
| Yes | -0.5 | -1.22 | -0.36 |
| **Type of work** |  |  |  |
| Unemployed |  |  |  |
| Casual | -0.49 | 0.67 | 0.51 |
| Self-employed | -0.41 | -0.32 | -0.21 |
| Government/private | 0.2 | 0.59 | -0.19 |
| **Percapita consumption expenditure** |  |  |  |
| Q1 (the lowest) |  |  |  |
| Q2 | -0.21 | 0.16 | 0.26 |
| Q3 | 2.36** | 0.37 | -0.02 |
| Q4 | -0.41 | -0.03 | 0.06 |
| Q5 (the highest) | -1.72* | -1.08 | -0.49 |
| **Residency** |  |  |  |
| Rural |  |  |  |
| Urban | 0.53 | -0.13 | -0.16 |
| **Island** |  |  |  |
| Java-Bali |  |  |  |
| Sumatra | 0.84 | -0.52 | 0.3 |
| Nusa Tenggara | 2.3** | 0.27 | 0.32 |
| Kalimantan | -0.33 | 0.62 | -0.06 |
| Sulawesi | -0.39 | -0.12 | -0.12 |
| **Tobacco consumption** |  |  |  |
| Never use tobacco |  |  |  |
| Former user | 0.42 | -0.11 | 0.24 |
| Light user | 1.37 | 0.52 | -0.35 |
| Moderate user | -0.81 | 0.6 | -0.49 |
| Heavy user | 0.82 | 0.65 | -0.04 |
| **BMI (kg/m2)** |  |  |  |
| Normal (18.5-23.0) |  |  |  |
| Underweight (<18.5) | -0.04 | -0.1 | 0.13 |
| Overweight (23.0 - <25.0) | 1.74* | 0.67 | 0.05 |
| Obesity (>= 30) | -0.76 | -0.49 | -0.12 |
| **Physical inactivity** |  |  |  |
| High |  |  |  |
| Moderate | 0.7 | -0.12 | 0.04 |
| Low | -0.36 | -0.28 | 0.17 |

* p<0.1; ** p<0.05; *** p<0.01

#### Table S7. Mean biases of covariates after matching using individual t-test (age group 60-69 vs 40-49)

| **Variable** | **Matching Algorithm** | | |
| --- | --- | --- | --- |
|  | Nearest neighbour with replacement | Nearest neighbour without replacement | Kernel |
| **Sex (%)** |  |  |  |
| Female |  |  |  |
| Male | -2.87*** | 1.31 | -2.21** |
| **Marital status (%)** |  |  |  |
| Not currently married |  |  |  |
| Currently Married | -1.98** | 0.05 | -1.3 |
| **Education (%)** |  |  |  |
| No education |  |  |  |
| Primary | 0.04 | 0.24 | -0.52 |
| Junior high school | 0.64 | -0.12 | -0.04 |
| Senior high school | 0.85 | 0.88 | 0.41 |
| Tertiary | 1.83* | 0.34 | 0.69 |
| **Ethnicity** |  |  |  |
| Javanese |  |  |  |
| Sundanese | 2.77*** | 0.22 | 0.22 |
| Others | -0.26 | 0.74 | -0.41 |
| **Had any health insurance** |  |  |  |
| No |  |  |  |
| Yes | 0.7 | -0.15 | 0.73 |
| **Type of work** |  |  |  |
| Unemployed |  |  |  |
| Casual | -1.4 | -0.15 | -0.68 |
| Self-employed | -1.92* | -0.88 | -1.16 |
| Government/private | 0.36 | 1.05 | 0.35 |
| **Percapita consumption expenditure** |  |  |  |
| Q1 (the lowest) |  |  |  |
| Q2 | 0.53 | 0.5 | -0.59 |
| Q3 | 0.32 | -0.14 | 0.15 |
| Q4 | 0.33 | 0.42 | 0.41 |
| Q5 (the highest) | 0.45 | -0.19 | 0.29 |
| **Residency** |  |  |  |
| Rural |  |  |  |
| Urban | 0.55 | 0.04 | -0.04 |
| **Island** |  |  |  |
| Java-Bali |  |  |  |
| Sumatra | 2.62*** | 0 | 0.96 |
| Nusa Tenggara | 0.07 | 0.39 | -0.32 |
| Kalimantan | 0.25 | 0.09 | -0.13 |
| Sulawesi | 1.23 | 0.6 | -0.36 |
| **Tobacco consumption** |  |  |  |
| Never use tobacco |  |  |  |
| Former user | 2.1** | 0.41 | 0.99 |
| Light user | -1.43 | 0.25 | -0.93 |
| Moderate user | -0.71 | 0.37 | -0.42 |
| Heavy user | -0.76 | 0.15 | -0.22 |
| **BMI (kg/m2)** |  |  |  |
| Normal (18.5-23.0) |  |  |  |
| Underweight (<18.5) | -2.6*** | -0.06 | -0.89 |
| Overweight (23.0 - <25.0) | 2.01* | 1.38 | 0.54 |
| Obesity (>= 30) | 0.78 | -1.01 | 0.83 |
| **Physical inactivity** |  |  |  |
| High |  |  |  |
| Moderate | -0.35 | -0.56 | 0.31 |
| Low | 1.2 | 0.26 | 0.92 |

* p<0.1; ** p<0.05; *** p<0.01

#### Table S8. Mean biases of covariates after matching using individual t-test (age group 70+ vs 40-49)

| **Variable** | **Matching Algorithm** | | |
| --- | --- | --- | --- |
|  | Nearest neighbour with replacement | Nearest neighbour without replacement | Kernel |
| **Sex (%)** |  |  |  |
| Female |  |  |  |
| Male | -3.09*** | -0.39 | -4.25*** |
| **Marital status (%)** |  |  |  |
| Not currently married |  |  |  |
| Currently Married | -0.47 | 0.85 | -0.74 |
| **Education (%)** |  |  |  |
| No education |  |  |  |
| Primary | 0.29 | -0.92 | -0.24 |
| Junior high school | 0.74 | -0.12 | -0.49 |
| Senior high school | 2.07** | 0.71 | -0.23 |
| Tertiary | 0.81 | -0.31 | 0.27 |
| **Ethnicity** |  |  |  |
| Javanese |  |  |  |
| Sundanese | 1.08 | -0.26 | 0.94 |
| Others | 0.48 | 1.01 | -1.74 |
| **Had any health insurance** |  |  |  |
| No |  |  |  |
| Yes | 2.32** | 0.22 | 0.47 |
| **Type of work** |  |  |  |
| Unemployed |  |  |  |
| Casual | -0.39 | -1.12 | -0.22 |
| Self-employed | -2.23** | 0 | -2.74*** |
| Government/private | 1.37 | 0.83 | -1.63 |
| **Percapita consumption expenditure** |  |  |  |
| Q1 (the lowest) |  |  |  |
| Q2 | -1.05 | 0.86 | -0.41 |
| Q3 | 1.55 | 0.28 | 2.33** |
| Q4 | 1.03 | 0.53 | -1 |
| Q5 (the highest) | -3.11*** | -0.91 | -1.47 |
| **Residency** |  |  |  |
| Rural |  |  |  |
| Urban | -0.20 | -0.38 | -1.91* |
| **Island** |  |  |  |
| Java-Bali |  |  |  |
| Sumatra | -1.77* | 0.49 | -0.04 |
| Nusa Tenggara | -0.21 | 0.1 | -1.82* |
| Kalimantan | 0.82 | 0 | 1.68* |
| Sulawesi | -1.56 | 0.77 | -0.39 |
| **Tobacco consumption** |  |  |  |
| Never use tobacco |  |  |  |
| Former user | -3.42*** | 0 | 0.28 |
| Light user | -2.47** | -0.65 | -2.19** |
| Moderate user | 0 | 0.52 | -1.4 |
| Heavy user | -0.52 | -0.36 | 0.45 |
| **BMI (kg/m2)** |  |  |  |
| Normal (18.5-23.0) |  |  |  |
| Underweight (<18.5) | 2.24** | 0.8 | 0.6 |
| Overweight (23.0 - <25.0) | -3.55*** | 0.33 | 0.55 |
| Obesity (>= 30) | -0.46 | 0.14 | -0.03 |
| **Physical inactivity** |  |  |  |
| High |  |  |  |
| Moderate | 0.89 | 0.47 | -0.35 |
| Low | 2.09** | 0 | 1.71* |

* p<0.1; ** p<0.05; *** p<0.01

#### Table S9. Mean biases of covariates after matching using individual t-test (former vs never use tobacco)

| **Variable** | **Matching Algorithm** | | |
| --- | --- | --- | --- |
|  | Nearest neighbour with replacement | Nearest neighbour without replacement | Kernel |
| **Age (%)** |  |  |  |
| 40 – 49 years |  |  |  |
| 50 – 59 years | 1.06 | 0.83 | 0.69 |
| 60 – 69 years | 0.61 | -0.12 | 0.31 |
| 70+ years | -0.92 | -0.99 | 0.27 |
| **Sex (%)** |  |  |  |
| Female |  |  |  |
| Male | -0.14 | -0.15 | 0.01 |
| **Marital status (%)** |  |  |  |
| Not currently married |  |  |  |
| Currently Married | 1.11 | 0.63 | -0.14 |
| **Education (%)** |  |  |  |
| No education |  |  |  |
| Primary | 1.33 | 0.79 | 0.38 |
| Junior high school | 2.26 | 0.39 | 0.24 |
| Senior high school | -0.41 | -0.43 | -0.24 |
| Tertiary | 0.48 | 0 | -0.33 |
| **Ethnicity** |  |  |  |
| Javanese |  |  |  |
| Sundanese | 0.08 | 0.25 | -0.31 |
| Others | 0.86 | 0.1 | 0.89 |
| **Had any health insurance** |  |  |  |
| No |  |  |  |
| Yes | 0.23 | 0.05 | -0.53 |
| **Type of work** |  |  |  |
| Unemployed |  |  |  |
| Casual | -0.08 | 0.49 | -0.16 |
| Self-employed | 0.86 | 0 | 0.47 |
| Government/private | 0.82 | 0.3 | -0.47 |
| **Percapita consumption expenditure** |  |  |  |
| Q1 (the lowest) |  |  |  |
| Q2 | -0.72 | 0.07 | -0.39 |
| Q3 | 2.13** | 0.26 | 0.18 |
| Q4 | 0.69 | -0.43 | 0.89 |
| Q5 (the highest) | 0.39 | 0.54 | 0.02 |
| **Residency** |  |  |  |
| Rural |  |  |  |
| Urban | -2.23** | -0.16 | -1.09 |
| **Island** |  |  |  |
| Java-Bali |  |  |  |
| Sumatra | 2.97*** | 1.04 | 2.13** |
| Nusa Tenggara | -0.34 | -0.46 | -0.5 |
| Kalimantan | -0.1 | -0.11 | -0.33 |
| Sulawesi | -0.51 | -0.32 | 0.13 |
| **BMI (kg/m2)** |  |  |  |
| Normal (18.5-23.0) |  |  |  |
| Underweight (<18.5) | -0.2 | -1.09 | -0.05 |
| Overweight (23.0 - <25.0) | 0.87 | 0.19 | -0.07 |
| Obesity (>= 30) | 0.66 | 0.97 | -0.49 |
| **Physical inactivity** |  |  |  |
| High |  |  |  |
| Moderate | 1.01 | 0.34 | 0.16 |
| Low | -0.7 | -0.66 | -0.08 |

* p<0.1; ** p<0.05; *** p<0.01

#### Table S10. Mean biases of covariates after matching using individual t-test (light user vs never use tobacco)

| **Variable** | **Matching Algorithm** | | |
| --- | --- | --- | --- |
|  | Nearest neighbour with replacement | Nearest neighbour without replacement | Kernel |
| **Age (%)** |  |  |  |
| 40 – 49 years |  |  |  |
| 50 – 59 years | -0.29 | -0.63 | -0.27 |
| 60 – 69 years | -0.59 | -1.05 | 0.05 |
| 70+ years | 1.03 | 0.33 | 0.18 |
| **Sex (%)** |  |  |  |
| Female |  |  |  |
| Male | -0.07 | -0.07 | -0.04 |
| **Marital status (%)** |  |  |  |
| Not currently married |  |  |  |
| Currently Married | -0.79 | 0.07 | -0.32 |
| **Education (%)** |  |  |  |
| No education |  |  |  |
| Primary | 1.01 | 0.22 | 0.48 |
| Junior high school | 1.9* | 0 | -0.37 |
| Senior high school | -1.35 | 0.05 | -0.81 |
| Tertiary | -0.73 | -0.37 | -0.45 |
| **Ethnicity** |  |  |  |
| Javanese |  |  |  |
| Sundanese | -1.2 | -1.32 | 0.57 |
| Others | -0.09 | -0.62 | 0.24 |
| **Had any health insurance** |  |  |  |
| No |  |  |  |
| Yes | -0.39 | 0.33 | -0.08 |
| **Type of work** |  |  |  |
| Unemployed |  |  |  |
| Casual | 2.48** | 0.94 | 0.79 |
| Self-employed | -0.97 | -0.66 | -0.36 |
| Government/private | 0.15 | 0.95 | 0.03 |
| **Percapita consumption expenditure** |  |  |  |
| Q1 (the lowest) |  |  |  |
| Q2 | 3.2*** | 1.15 | -0.02 |
| Q3 | -1.26 | 0.4 | 0.09 |
| Q4 | -0.17 | -0.87 | 0.3 |
| Q5 (the highest) | -2.22** | -0.89 | -0.21 |
| **Residency** |  |  |  |
| Rural |  |  |  |
| Urban | -1.81* | -0.24 | -0.94 |
| **Island** |  |  |  |
| Java-Bali |  |  |  |
| Sumatra | -0.16 | -0.24 | 0.41 |
| Nusa Tenggara | 0.9 | 0.29 | 0.38 |
| Kalimantan | 0.53 | 0.13 | -0.55 |
| Sulawesi | -0.64 | -0.11 | -0.07 |
| **BMI (kg/m2)** |  |  |  |
| Normal (18.5-23.0) |  |  |  |
| Underweight (<18.5) | 1.31 | 0 | 0.9 |
| Overweight (23.0 - <25.0) | -0.85 | -0.25 | -0.63 |
| Obesity (>= 30) | -0.39 | -0.05 | -1.24 |
| **Physical inactivity** |  |  |  |
| High |  |  |  |
| Moderate | 1.4 | -0.11 | -0.52 |
| Low | -2.28** | -0.67 | -0.24 |

* p<0.1; ** p<0.05; *** p<0.01

#### Table S11. Mean biases of covariates after matching using individual t-test (moderate user vs never use tobacco)

| **Variable** | **Matching Algorithm** | | |
| --- | --- | --- | --- |
|  | Nearest neighbour with replacement | Nearest neighbour without replacement | Kernel |
| **Age (%)** |  |  |  |
| 40 – 49 years |  |  |  |
| 50 – 59 years | 0.78 | 1.3 | 0.61 |
| 60 – 69 years | 0.08 | 0.28 | -0.38 |
| 70+ years | -0.71 | -2.0** | -1.93* |
| **Sex (%)** |  |  |  |
| Female |  |  |  |
| Male | -0.11 | -0.06 | 0.04 |
| **Marital status (%)** |  |  |  |
| Not currently married |  |  |  |
| Currently Married | 3.2 | 0.42 | 1.72* |
| **Education (%)** |  |  |  |
| No education |  |  |  |
| Primary | 0.58 | 0.25 | 0.69 |
| Junior high school | 0.87 | 0.75 | -0.36 |
| Senior high school | 0.92 | 0.33 | -0.5 |
| Tertiary | -0.65 | 0.23 | -0.69 |
| **Ethnicity** |  |  |  |
| Javanese |  |  |  |
| Sundanese | 0.88 | -0.13 | 0.74 |
| Others | 0.94 | -0.25 | -0.28 |
| **Had any health insurance** |  |  |  |
| No |  |  |  |
| Yes | 3.19 | -1.03 | 1.29 |
| **Type of work** |  |  |  |
| Unemployed |  |  |  |
| Casual | 0.24 | 0.53 | 1.51 |
| Self-employed | 0.21 | -0.37 | 0.35 |
| Government/private | 1.35 | 0 | 0.91 |
| **Percapita consumption expenditure** |  |  |  |
| Q1 (the lowest) |  |  |  |
| Q2 | -2.97*** | 0.88 | -0.35 |
| Q3 | 0.44 | -0.56 | -0.95 |
| Q4 | 1.62 | -0.41 | 0.83 |
| Q5 (the highest) | 0.16 | 0.73 | -0.09 |
| **Residency** |  |  |  |
| Rural |  |  |  |
| Urban | 2.05** | 0.67 | 0.08 |
| **Island** |  |  |  |
| Java-Bali |  |  |  |
| Sumatra | 2.39** | -0.11 | 2.19** |
| Nusa Tenggara | 0.23 | 0.08 | -1.15 |
| Kalimantan | -3.68*** | -0.89 | -1.83* |
| Sulawesi | 0.45 | 0.19 | -0.35 |
| **BMI (kg/m2)** |  |  |  |
| Normal (18.5-23.0) |  |  |  |
| Underweight (<18.5) | 0.65 | -0.07 | 0.45 |
| Overweight (23.0 - <25.0) | 0.89 | 0.93 | -0.88 |
| Obesity (>= 30) | -0.48 | 1.17 | -0.88 |
| **Physical inactivity** |  |  |  |
| High |  |  |  |
| Moderate | -1.45 | -0.71 | 0.2 |
| Low | -0.04 | 1.22 | -2.08** |

* p<0.1; ** p<0.05; *** p<0.01

#### Table S12. Mean biases of covariates after matching using individual t-test (heavy user vs never use tobacco)

| **Variable** | **Matching Algorithm** | | |
| --- | --- | --- | --- |
|  | Nearest neighbour with replacement | Nearest neighbour without replacement | Kernel |
| **Age (%)** |  |  |  |
| 40 – 49 years |  |  |  |
| 50 – 59 years | -0.31 | -0.13 | 0.24 |
| 60 – 69 years | 0.93 | -0.49 | 0.3 |
| 70+ years | -1.54 | 0.39 | -1.11 |
| **Sex (%)** |  |  |  |
| Female |  |  |  |
| Male | 0 | 0 | 0.24 |
| **Marital status (%)** |  |  |  |
| Not currently married |  |  |  |
| Currently Married | 0.74 | -0.18 | 0.67 |
| **Education (%)** |  |  |  |
| No education |  |  |  |
| Primary | -0.57 | 0.35 | 0.06 |
| Junior high school | 0.56 | -0.09 | 0.18 |
| Senior high school | -0.26 | 0.21 | 0 |
| Tertiary | -0.12 | -0.25 | 0.01 |
| **Ethnicity** |  |  |  |
| Javanese |  |  |  |
| Sundanese | 0.18 | 0.7 | 0.67 |
| Others | -0.05 | -0.06 | 0.56 |
| **Had any health insurance** |  |  |  |
| No |  |  |  |
| Yes | 0.65 | 0.29 | 0.53 |
| **Type of work** |  |  |  |
| Unemployed |  |  |  |
| Casual | -0.16 | -0.43 | 0.29 |
| Self-employed | 1.25 | 0.53 | 0.23 |
| Government/private | -0.68 | -0.2 | 0.35 |
| **Percapita consumption expenditure** |  |  |  |
| Q1 (the lowest) |  |  |  |
| Q2 | 0.07 | 0.23 | -0.1 |
| Q3 | -0.2 | 0.43 | -0.25 |
| Q4 | -0.41 | -0.95 | 0.49 |
| Q5 (the highest) | -0.8 | -0.77 | 0.1 |
| **Residency** |  |  |  |
| Rural |  |  |  |
| Urban | -0.22 | -0.18 | 0.49 |
| **Island** |  |  |  |
| Java-Bali |  |  |  |
| Sumatra | 0.61 | -0.63 | 0.6 |
| Nusa Tenggara | -0.77 | -0.41 | -0.27 |
| Kalimantan | -1.26 | 0.25 | -0.69 |
| Sulawesi | 1.5 | -0.28 | -0.13 |
| **BMI (kg/m2)** |  |  |  |
| Normal (18.5-23.0) |  |  |  |
| Underweight (<18.5) | -1.27 | -0.28 | -0.43 |
| Overweight (23.0 - <25.0) | 2.06** | 0.86 | 0.5 |
| Obesity (>= 30) | -1.45 | -0.73 | -0.08 |
| **Physical inactivity** |  |  |  |
| High |  |  |  |
| Moderate | -0.38 | 0 | 0.21 |
| Low | -0.39 | 0.18 | -0.79 |

* p<0.1; ** p<0.05; *** p<0.01

#### Table S13. Mean biases of covariates after matching using individual t-test (overweight vs normal BMI)

| **Variable** | **Matching Algorithm** | | |
| --- | --- | --- | --- |
|  | Nearest neighbour with replacement | Nearest neighbour without replacement | Kernel |
| **Age (%)** |  |  |  |
| 40 – 49 years |  |  |  |
| 50 – 59 years | 2.05 | 0.35 | -0.02 |
| 60 – 69 years | -0.13 | -0.59 | -0.41 |
| 70+ years | -0.7 | -0.06 | 0.55 |
| **Sex (%)** |  |  |  |
| Female |  |  |  |
| Male | 0.28 | 0.57 | 0.16 |
| **Marital status (%)** |  |  |  |
| Not currently married |  |  |  |
| Currently Married | 0.64 | 0.48 | 0.33 |
| **Education (%)** |  |  |  |
| No education |  |  |  |
| Primary | -0.29 | -0.4 | -0.4 |
| Junior high school | 2.24** | 0.43 | -0.16 |
| Senior high school | -1.75* | 0.43 | 0.18 |
| Tertiary | 1.02 | 0.54 | 0.71 |
| **Ethnicity** |  |  |  |
| Javanese |  |  |  |
| Sundanese | 2.57** | 1.06 | -0.07 |
| Others | 2.25** | 0.87 | 0.05 |
| **Had any health insurance** |  |  |  |
| No |  |  |  |
| Yes | -0.69 | 0.44 | 0.23 |
| **Type of work** |  |  |  |
| Unemployed |  |  |  |
| Casual | 0.17 | -0.04 | -0.22 |
| Self-employed | 0.71 | -0.39 | -0.1 |
| Government/private | 1.2 | 0.64 | 0.41 |
| **Percapita consumption expenditure** |  |  |  |
| Q1 (the lowest) |  |  |  |
| Q2 | -0.39 | -0.43 | -0.5 |
| Q3 | 0.46 | -0.5 | -0.01 |
| Q4 | -1.02 | 0.47 | 0.13 |
| Q5 (the highest) | 0.73 | 0.31 | 0.34 |
| **Residency** |  |  |  |
| Rural |  |  |  |
| Urban | -0.51 | 0.35 | 0.27 |
| **Island** |  |  |  |
| Java-Bali |  |  |  |
| Sumatra | 0.49 | 0.11 | 0.09 |
| Nusa Tenggara | 1.17 | 0.82 | 0.17 |
| Kalimantan | 0.58 | -0.14 | 0.08 |
| Sulawesi | 0.85 | 0.77 | 0.17 |
| **Tobacco consumption** |  |  |  |
| Never use tobacco |  |  |  |
| Former user | 0.47 | 0.7 | 0.01 |
| Light user | 1.29 | 1.22 | 0.18 |
| Moderate user | -0.6 | 0.16 | 0.15 |
| Heavy user | 0.91 | 0.21 | -0.01 |
| **Physical inactivity** |  |  |  |
| High |  |  |  |
| Moderate | -0.03 | -0.28 | 0.03 |
| Low | 0.87 | 0.58 | 0.06 |

* p<0.1; ** p<0.05; *** p<0.01

#### Table S14. Mean biases of covariates after matching using individual t-test (obesity vs normal BMI)

| **Variable** | **Matching Algorithm** | | |
| --- | --- | --- | --- |
|  | Nearest neighbour with replacement | Nearest neighbour without replacement | Kernel |
| **Age (%)** |  |  |  |
| 40 – 49 years |  |  |  |
| 50 – 59 years | -1.75* | -0.81 | 0.15 |
| 60 – 69 years | -0.25 | 0.55 | -0.55 |
| 70+ years | 0.84 | 0.45 | 0.65 |
| **Sex (%)** |  |  |  |
| Female |  |  |  |
| Male | 0.45 | 0.6 | 0.87 |
| **Marital status (%)** |  |  |  |
| Not currently married |  |  |  |
| Currently Married | 1.65* | -1.05 | 1.47 |
| **Education (%)** |  |  |  |
| No education |  |  |  |
| Primary | 1.44 | -0.41 | -0.5 |
| Junior high school | 1.45 | 0.21 | 0.13 |
| Senior high school | -1.48 | -0.07 | -0.91 |
| Tertiary | -0.14 | -0.34 | 1.61 |
| **Ethnicity** |  |  |  |
| Javanese |  |  |  |
| Sundanese | 1.29 | -1.02 | 0.66 |
| Others | 1.12 | 0.69 | 0.13 |
| **Had any health insurance** |  |  |  |
| No |  |  |  |
| Yes | -0.11 | -1.03 | -0.11 |
| **Type of work** |  |  |  |
| Unemployed |  |  |  |
| Casual | -0.82 | 0.64 | -1.16 |
| Self-employed | -0.65 | -0.25 | 0.83 |
| Government/private | -0.9 | -0.26 | -0.11 |
| **Percapita consumption expenditure** |  |  |  |
| Q1 (the lowest) |  |  |  |
| Q2 | -0.81 | 0.27 | -1.1 |
| Q3 | -0.37 | -0.34 | -0.35 |
| Q4 | 1.45 | -0.56 | -0.16 |
| Q5 (the highest) | -0.07 | -0.5 | 1.93 |
| **Residency** |  |  |  |
| Rural |  |  |  |
| Urban | 0.79 | -0.66 | 0 |
| **Island** |  |  |  |
| Java-Bali |  |  |  |
| Sumatra | -0.34 | 1.14 | 0.16 |
| Nusa Tenggara | 0.19 | 0.29 | 0.18 |
| Kalimantan | 0.86 | 0.73 | -0.34 |
| Sulawesi | 1.72* | -0.46 | 0.02 |
| **Tobacco consumption** |  |  |  |
| Never use tobacco |  |  |  |
| Former user | 0 | -0.56 | -0.06 |
| Light user | 0 | 1.37 | 0.28 |
| Moderate user | 0.39 | 0.67 | 0.48 |
| Heavy user | 0.35 | 0.6 | 0.29 |
| **Physical inactivity** |  |  |  |
| High |  |  |  |
| Moderate | -0.21 | 0.59 | -0.16 |
| Low | -0.18 | -1 | 0.35 |

* p<0.1; ** p<0.05; *** p<0.01

#### Table S15. Mean biases of covariates after matching using individual t-test (underweight vs normal BMI)

| **Variable** | **Matching Algorithm** | | |
| --- | --- | --- | --- |
|  | Nearest neighbour with replacement | Nearest neighbour without replacement | Kernel |
| **Age (%)** |  |  |  |
| 40 – 49 years |  |  |  |
| 50 – 59 years | 0.56 | 0.47 | 0.1 |
| 60 – 69 years | -1.3 | -0.57 | 0.11 |
| 70+ years | 1.2 | 0.62 | 0.01 |
| **Sex (%)** |  |  |  |
| Female |  |  |  |
| Male | 1.61 | 0.2 | 0.3 |
| **Marital status (%)** |  |  |  |
| Not currently married |  |  |  |
| Currently Married | -0.04 | 0.17 | 0.07 |
| **Education (%)** |  |  |  |
| No education |  |  |  |
| Primary | -0.28 | -0.19 | 0 |
| Junior high school | 1.51 | 1.52 | -0.12 |
| Senior high school | -1.15 | -0.48 | -0.2 |
| Tertiary | 0.14 | 0.87 | -0.19 |
| **Ethnicity** |  |  |  |
| Javanese |  |  |  |
| Sundanese | -0.5 | -0.69 | 0.14 |
| Others | -0.47 | 0.32 | -0.16 |
| **Had any health insurance** |  |  |  |
| No |  |  |  |
| Yes | -0.12 | -0.24 | -0.09 |
| **Type of work** |  |  |  |
| Unemployed |  |  |  |
| Casual | -0.44 | -0.69 | -0.01 |
| Self-employed | 0.9 | 0.91 | 0.07 |
| Government/private | -0.18 | -0.6 | 0.16 |
| **Percapita consumption expenditure** |  |  |  |
| Q1 (the lowest) |  |  |  |
| Q2 | 0.67 | 0.68 | -0.03 |
| Q3 | 0.36 | 0.41 | 0.11 |
| Q4 | -0.83 | -0.28 | 0.07 |
| Q5 (the highest) | 1.37 | 0.6 | -0.07 |
| **Residency** |  |  |  |
| Rural |  |  |  |
| Urban | 0.4 | 0.12 | -0.3 |
| **Island** |  |  |  |
| Java-Bali |  |  |  |
| Sumatra | -0.65 | -0.3 | -0.33 |
| Nusa Tenggara | -0.25 | 0.4 | -0.36 |
| Kalimantan | 0.5 | 0.18 | 0.05 |
| Sulawesi | 0.09 | 0.18 | -0.03 |
| **Tobacco consumption** |  |  |  |
| Never use tobacco |  |  |  |
| Former user | 0.13 | 0.4 | 0.21 |
| Light user | 0.31 | -0.06 | 0.23 |
| Moderate user | 0.83 | 0.19 | -0.39 |
| Heavy user | 0.74 | 1.08 | 0.24 |
| **Physical inactivity** |  |  |  |
| High |  |  |  |
| Moderate | 0.98 | 0.97 | -0.16 |
| Low | -0.09 | 0.12 | 0.16 |

* p<0.1; ** p<0.05; *** p<0.01

#### Table S16. Mean biases of covariates after matching using individual t-test (low vs high physical activity)

| **Variable** | **Matching Algorithm** | | |
| --- | --- | --- | --- |
|  | Nearest neighbour with replacement | Nearest neighbour without replacement | Kernel |
| **Age (%)** |  |  |  |
| 40 – 49 years |  |  |  |
| 50 – 59 years | -0.09 | 0.39 | -0.03 |
| 60 – 69 years | -0.46 | 0.45 | -0.7 |
| 70+ years | 1.59 | -2.51** | 2.03 |
| **Sex (%)** |  |  |  |
| Female |  |  |  |
| Male | 2.05** | 0.62 | 0.33 |
| **Marital status (%)** |  |  |  |
| Not currently married |  |  |  |
| Currently Married | -0.81 | 1.35 | -1.66* |
| **Education (%)** |  |  |  |
| No education |  |  |  |
| Primary | 0.1 | 0.39 | 0.22 |
| Junior high school | 1.52 | -0.35 | -0.33 |
| Senior high school | -0.84 | -0.26 | -1.87* |
| Tertiary | -1.6 | 0.36 | -2.33** |
| **Ethnicity** |  |  |  |
| Javanese |  |  |  |
| Sundanese | 0.19 | 0.81 | 0.04 |
| Others | 2.21** | 0.74 | 1.38 |
| **Had any health insurance** |  |  |  |
| No |  |  |  |
| Yes | -1.08 | 0.67 | -1.11 |
| **Type of work** |  |  |  |
| Unemployed |  |  |  |
| Casual | -1.13 | 0.33 | 0.25 |
| Self-employed | -1.85* | 0.44 | -0.88 |
| Government/private | 0.91 | -0.3 | 0.15 |
| **Percapita consumption expenditure** |  |  |  |
| Q1 (the lowest) |  |  |  |
| Q2 | 0.56 | -0.35 | 0.4 |
| Q3 | 3.06*** | 0.19 | 0.92 |
| Q4 | -0.43 | 0.66 | -0.24 |
| Q5 (the highest) | -0.95 | 0.1 | -2.22** |
| **Residency** |  |  |  |
| Rural |  |  |  |
| Urban | -4.65*** | 0.23 | -3.62*** |
| **Island** |  |  |  |
| Java-Bali |  |  |  |
| Sumatra | -1.88* | -0.37 | 0.37 |
| Nusa Tenggara | 2.7*** | 1.51 | 0.73 |
| Kalimantan | 2.12** | 0.58 | 0.22 |
| Sulawesi | -0.24 | -0.89 | 0.58 |
| **Tobacco consumption** |  |  |  |
| Never use tobacco |  |  |  |
| Former user | 1.83* | -0.61 | 1.47 |
| Light user | -0.91 | 0.47 | -1.64 |
| Moderate user | 1.72* | 0.41 | 0.91 |
| Heavy user | 1 | 0.46 | -0.57 |
| **BMI (kg/m2)** |  |  |  |
| Normal (18.5-23.0) |  |  |  |
| Underweight (<18.5) | 1.74* | -0.77 | 0.88 |
| Overweight (23.0 - <25.0) | 1.89* | 0.58 | 1.5 |
| Obesity (>= 30) | -1.82* | -0.35 | -1.99** |

* p<0.1; ** p<0.05; *** p<0.01

#### Table S17. Mean biases of covariates after matching using individual t-test (moderate vs high physical activity)

| **Variable** | **Matching Algorithm** | | |
| --- | --- | --- | --- |
|  | Nearest neighbour with replacement | Nearest neighbour without replacement | Kernel |
| **Age (%)** |  |  |  |
| 40 – 49 years |  |  |  |
| 50 – 59 years | 0.55 | 0.17 | 0.01 |
| 60 – 69 years | 1.16 | 0.44 | 0.11 |
| 70+ years | -0.01 | -0.91 | 0.4 |
| **Sex (%)** |  |  |  |
| Female |  |  |  |
| Male | 1.9* | 1.16 | 0.74 |
| **Marital status (%)** |  |  |  |
| Not currently married |  |  |  |
| Currently Married | 0.22 | 0.61 | -0.08 |
| **Education (%)** |  |  |  |
| No education |  |  |  |
| Primary | 0.96 | 0.61 | 0.34 |
| Junior high school | -0.18 | -0.37 | -0.02 |
| Senior high school | -1.75* | -0.17 | -0.7 |
| Tertiary | -0.26 | -0.7 | 0.11 |
| **Ethnicity** |  |  |  |
| Javanese |  |  |  |
| Sundanese | 0.03 | -0.53 | 0.82 |
| Others | 1.33 | -0.19 | -0.41 |
| **Had any health insurance** |  |  |  |
| No |  |  |  |
| Yes | -1.57 | 0.19 | -0.06 |
| **Type of work** |  |  |  |
| Unemployed |  |  |  |
| Casual | -0.07 | 0.56 | -0.34 |
| Self-employed | 0.45 | 0.51 | -0.62 |
| Government/private | -1.17 | -0.32 | -0.05 |
| **Percapita consumption expenditure** |  |  |  |
| Q1 (the lowest) |  |  |  |
| Q2 | -0.74 | 0.03 | -0.16 |
| Q3 | -0.21 | -0.75 | 0.12 |
| Q4 | -0.21 | 0.47 | -0.21 |
| Q5 (the highest) | 0.44 | -0.58 | 0 |
| **Residency** |  |  |  |
| Rural |  |  |  |
| Urban | -1.14 | -0.73 | -0.52 |
| **Island** |  |  |  |
| Java-Bali |  |  |  |
| Sumatra | 1.16 | -0.07 | -0.58 |
| Nusa Tenggara | -1.12 | -0.4 | 0.02 |
| Kalimantan | 1.04 | 0.06 | 0.28 |
| Sulawesi |  |  | 0.69 |
| **Tobacco consumption** |  |  |  |
| Never use tobacco |  |  |  |
| Former user | 0.76 | -0.16 | 0.29 |
| Light user | -0.05 | 0.24 | -0.02 |
| Moderate user | 1.6 | 1.19 | 0.68 |
| Heavy user | 0.29 | 0 | 0.03 |
| **BMI (kg/m2)** |  |  |  |
| Normal (18.5-23.0) |  |  |  |
| Underweight (<18.5) | 1.35 | 0.14 | 0.15 |
| Overweight (23.0 - <25.0) | 0.32 | 0.31 | -0.18 |
| Obesity (>= 30) | -0.52 | -1.19 | -0.32 |

* p<0.1; ** p<0.05; *** p<0.01

#### Table S18. The ATT of the number of chronic conditions across different matching algorithms

| **Risk factors** | **Number of chronic condition** | | | | | | |
| --- | --- | --- | --- | --- | --- | --- | --- |
|  | **Kernel** | | **Nearest Neighbour with replacement** | | | **Nearest Neighbour without replacement** | |
|  | ATT (95% CI) | % relative change to control | | ATT (95% CI) | % relative change to control | ATT (95% CI) | % relative change to control |
| **BMI (ref Normal BMI)** | |  | |  |  |  |  |
| Overweight | 0.13 (0.08, 0.19)*** | 17% | | 0.13 (0.05, 0.20)*** | 16% | 0.11 (0.05, 0.16)*** | 13% |
| Obesity | 0.29 (0.24, 0.34)*** | 35% | | 0.31 (0.23, 0.39)*** | 38% | 0.31 (0.25, 0.36)*** | 38% |
| Underweight | -0.1 (-0.16, -0.05)*** | -11% | | -0.10 (-0.19, -0.01)** | -11% | -0.09 (-0.17, -0.01)** | -10% |
| **Smoking (ref never use)** | |  | |  |  |  |  |
| Former user | 0.25 (0.15, 0.36)*** | 24% | | 0.30 (0.17, 0.43)*** | 30% | 0.25 (0.15, 0.35)*** | 24% |
| Light user | -0.07 (-0.15, 0.01)* | -8% | | -0.02 (-0.15, 0.11) | -3% | -0.06 (-0.15, 0.02) | -7% |
| Moderate user | -0.14 (-0.22, -0.07)*** | -17% | | -0.12 (-0.22, -0.02)** | -15% | -0.09 (-0.17, -0.02)** | -11% |
| Heavy user | -0.08 (-0.18, 0.005)* | -10% | | -0.13 (-0.29 0.03) | -15% | -0.1 (-0.21, 0.004)* | -13% |
| **PA (ref High PA)** |  |  | |  |  |  |  |
| Low PA | 0.006 (-0.04, 0.05) | 1% | | 0.02 (-0.04, 0.09) | 2% | -0.0007 (-0.05, 0.05) | 0% |
| Moderate PA | 0.04 (-0.002, 0.09)* | 5% | | 0.08 (0.005, 0.15)** | 8% | 0.04 (-0.01, 0.09) | 5% |
| **Ageing (ref age 40-49)** | |  | |  |  |  |  |
| Age 50-59 | 0.29 (0.24, 0.33)*** | 40% | | 0.26 (0.19, 0.32)*** | 34% | 0.28 (0.24, 0.33)*** | 39% |
| Age 60-69 | 0.48 (0.42, 0.55)*** | 68% | | 0.49 (0.41, 0.58)*** | 70% | 0.48 (0.40, 0.56)*** | 69% |
| Age 70+ | 0.46 (0.25, 0.66)*** | 60% | | 0.35 (0.12, 0.59)*** | 41% | 0.52 (0.41, 0.62)*** | 78% |

* p<0.1; ** p<0.05; *** p<0.01

#### Table S19. The ATT of multimorbidity presence across different matching algorithms

| **Risk factors** | **Presence of multimorbidity** | | | | | | | |  |
| --- | --- | --- | --- | --- | --- | --- | --- | --- | --- |
|  | **Kernel** | | **Nearest Neighbour with replacement** | | | **Nearest Neighbour without replacement** | | |  |
|  | ATT (95% CI) | % relative change to control | | ATT (95% CI) | % relative change to control | | ATT (95% CI) | % relative change to control | |
| **BMI (ref Normal BMI)** | |  | |  |  | |  |  | |
| Overweight | 0.03 (0.01, 0.06)*** | 18% | | 0.03 (-0.01, 0.06) | 14% | | 0.03 (0.003, 0.05)** | 15% | |
| Obesity | 0.09 (0.07, 0.11)*** | 47% | | 0.09 (0.06, 0.12)*** | 45% | | 0.09 (0.07, 0.11)*** | 51% | |
| Underweight | -0.008 (-0.03, 0.02) | -4% | | -0.01 (-0.05, 0.03) | -4% | | -0.007 (-0.04, 0.02) | -1% | |
| **Smoking (ref never use)** | |  | |  |  | |  |  | |
| Former user | 0.09 (0.04, 0.13)*** | 36% | | 0.10 (0.04, 0.15)*** | 41% | | 0.07 (0.03, 0.12)*** | 30% | |
| Light user | -0.04 (-0.07, -0.01)** | -19% | | -0.03 (-0.07, 0.02) | -14% | | -0.04 (-0.07, -0.002) | -18% | |
| Moderate user | -0.06 (-0.08, -0.03)*** | -33% | | -0.05 (-0.10, -0.007)** | -29% | | -0.04 (-0.07, -0.006)** | -20% | |
| Heavy user | -0.05 (-0.08, -0.01)** | -28% | | -0.06 (-0.12, -0.001)** | -32% | | -0.05 (-0.09, -0.01)** | -26% | |
| **PA (ref High PA)** | |  | |  |  | |  |  | |
| Low PA | 0.004 (-0.02, 0.03) | 2% | | 0.006 (-0.02, 0.03) | 3% | | 0.002 (-0.02, 0.02) | 1% | |
| Moderate PA | 0.02 (0.001, 0.04)** | 9% | | 0.03 (-0.003, 0.05)* | 12% | | 0.02 (-0.004, 0.04) | 8% | |
| **Ageing (ref age 40-49)** | |  | |  |  | |  |  | |
| Age 50-59 | 0.09 (0.07, 0.11)*** | 61% | | 0.08 (0.05, 0.11)*** | 50% | | 0.09 (0.07, 0.11)*** | 64% | |
| Age 60-69 | 0.16 (0.13, 0.18)*** | 114% | | 0.16 (0.13, 0.19)*** | 123% | | 0.15 (0.13, 0.18)*** | 106% | |
| Age 70+ | 0.14 (0.08, 0.20)*** | 102% | | 0.11 (0.03, 0.18)*** | 61% | | 0.13 (0.09 0.17)*** | 93% | |

* p<0.1; ** p<0.05; *** p<0.01

#### Table S20. The ATT of the number of outpatient visits across different matching algorithms

| **Risk factors** | **Number of outpatient visits** | | | | | | | |  |
| --- | --- | --- | --- | --- | --- | --- | --- | --- | --- |
|  | **Kernel** | | **Nearest Neighbour with replacement** | | | **Nearest Neighbour without replacement** | | |  |
|  | ATT (95% CI) | % relative change to control | | ATT (95% CI) | % relative change to control | | ATT (95% CI) | % relative change to control | |
| **BMI (ref Normal BMI)** | |  | |  |  | |  |  | |
| Overweight | 0.007 (-0.05, 0.07) | 2% | | -0.001 (-0.09, 0.08) | 0% | | 0.01 (-0.06, 0.09) | 4% | |
| Obesity | 0.04 (-0.01, 0.09) | 10% | | -0.006 (-0.07, 0.06) | -1% | | 0.03 (-0.03, 0.09) | 8% | |
| Underweight | 0.08 (0.01, 0.16)** | 23% | | 0.10 (0.001, 0.19)** | 26% | | 0.08 (-0.003, 0.16)* | 22% | |
| **Smoking (ref never use)** | |  | |  |  | |  |  | |
| Former user | 0.18 (0.05, 0.30)*** | 43% | | 0.11 (-0.05, 0.28) | 23% | | 0.13 (0.02, 0.24)** | 28% | |
| Light user | -0.09 (-0.18, 0.002)* | -24% | | 0.02 (-0.11, 0.14) | 6% | | -0.08 (-0.18, 0.02) | -22% | |
| Moderate user | -0.12 (-0.20, -0.05)*** | -35% | | -0.08 (-0.17, 0.01)* | -27% | | -0.16 (-0.25, -0.08)*** | -41% | |
| Heavy user | -0.09 (-0.19, 0.007)* | -26% | | -0.07 (-0.28, 0.14) | -21% | | -0.07 (-0.20, 0.07) | -22% | |
| **PA (ref High PA)** | |  | |  |  | |  |  | |
| Low PA | 0.10 (0.05, 0.15)*** | 30% | | 0.11 (0.06, 0.16)*** | 32% | | 0.06 (-0.004, 0.13) | 18% | |
| Moderate PA | -0.001 (-0.05, 0.05) | 0% | | -0.003 (-0.09, 0.08) | -1% | | -0.01 (-0.07, 0.05) | -2% | |
| **Ageing (ref age 40-49)** | |  | |  |  | |  |  | |
| Age 50-59 | 0.07 (0.02, 0.11)*** | 20% | | 0.004 (-0.08, 0.09) | 1% | | 0.04 (-0.03, 0.10) | 10% | |
| Age 60-69 | 0.11 (0.03, 0.18)*** | 30% | | 0.08 (-0.02, 0.18) | 21% | | 0.05 (-0.02, 0.12) | 13% | |
| Age 70+ | 0.05 (-0.13, 0.24) | 13% | | -0.004 (-0.28, 0.27) | -1% | | 0.14 (0.02, 0.25)** | 40% | |

* p<0.1; ** p<0.05; *** p<0.01

#### Table S21. The ATT of the number of inpatient visits across different matching algorithms

| **Risk factors** | **Number of inpatient visits** | | | | | | | |  |
| --- | --- | --- | --- | --- | --- | --- | --- | --- | --- |
|  | **Kernel** | | **Nearest Neighbour with replacement** | | | **Nearest Neighbour without replacement** | | |  |
|  | ATT (95% CI) | % relative change to control | | ATT (95% CI) | % relative change to control | | ATT (95% CI) | % relative change to control | |
| **BMI (ref Normal BMI)** | |  | |  |  | |  |  | |
| Overweight | 0.006 (-0.01, 0.03) | 10% | | 0.008 (-0.02, 0.04) | 15% | | 0.01 (-0.01, 0.03) | 19% | |
| Obesity | -0.006 (-0.02, 0.01) | -10% | | 0.005 (-0.02, 0.03) | 10% | | -0.007 (-0.03, 0.01) | -13% | |
| Underweight | 0.02 (-0.02, 0.05)% | 27% | | 0.008 (-0.04, 0.06) | 12% | | 0.02 (-0.02, 0.06) | 34% | |
| **Smoking (ref never use)** | |  | |  |  | |  |  | |
| Former user | 0.05 (0.01, 0.09)*** | 68% | | 0.04 (-0.02, 0.10) | 44% | | 0.05 (0.01, 0.09)** | 60% | |
| Light user | -0.04 (-0.06, -0.01)*** | -70% | | -0.03 (-0.07, 0.005)* | -60% | | -0.02 (-0.05, -0.002)** | -50% | |
| Moderate user | -0.02 (-0.04, -0.002)** | -40% | | -0.02 (-0.05, 0.01) | -40% | | -0.02 (-0.05, 0.004)* | -41% | |
| Heavy user | -0.02 (-0.04, 0.003)* | -40% | | -0.06 (-0.1, -0.02)*** | -64% | | -0.03 (-0.06, 0.002)* | -48% | |
| **PA (ref High PA)** | |  | |  |  | |  |  | |
| Low PA | 0.01 (-0.02, 0.04) | 17% | | 0.02 (-0.02, 0.05) | 25% | | 0.02 (0.008, 0.04)*** | 51% | |
| Moderate PA | -0.01 (-0.03, 0.01) | -15% | | -0.004 (-0.04, 0.03) | -8% | | -0.005 (-0.02, 0.01) | -10% | |
| **Ageing (ref age 40-49)** | |  | |  |  | |  |  | |
| Age 50-59 | 0.02 (0.004, 0.03)** | 51% | | 0.02 (-0.0003, 0.05)* | 68% | | 0.01 (-0.009, 0.03) | 28% | |
| Age 60-69 | 0.02 (-0.002, 0.05)* | 39% | | 0.04 (0.004, 0.07)** | 90% | | 0.02 (-0.01, 0.05) | 45% | |
| Age 70+ | 0.05 (0.005, 0.09)** | 85% | | 0.07 (0.02, 0.13)** | 216% | | -0.003 (-0.05, 0.05) | -3% | |

* p<0.1; ** p<0.05; *** p<0.01

#### Table S22. The ATT of CHE >25% of total household expenditure across different matching algorithms

| **Risk factors** | **CHE >25% of total household expenditure** | | | | | | | |  |
| --- | --- | --- | --- | --- | --- | --- | --- | --- | --- |
|  | **Kernel** | | **Nearest Neighbour with replacement** | | | **Nearest Neighbour without replacement** | | |  |
|  | ATT (95% CI) | % relative change to control | | ATT (95% CI) | % relative change to control | | ATT (95% CI) | % relative change to control | |
| **BMI (ref Normal BMI)** | |  | |  |  | |  |  | |
| Overweight | -0.007 (-0.01, -0.002)** | -50% | | -0.004 (-0.01, 0.004) | -40% | | -0.005 (-0.01, 0.002) | -42% | |
| Obesity | -0.003 (-0.01, 0.002) | -21% | | -0.006 (-0.01, 0.002) | -34% | | -0.001 (-0.008, 0.005) | -10% | |
| Underweight | -0.001 (-0.008, 0.006) | -8% | | -0.003 (-0.01, 0.007) | -20% | | -0.002 (-0.01, 0.01) | -11% | |
| **Smoking (ref never use)** | |  | |  |  | |  |  | |
| Former user | 0.01 (-0.002, 0.02) | 85% | | 0.01 (-0.01, 0.03) | 109% | | 0.01 (-0.002, 0.02) | 68% | |
| Light user | 0.003 (-0.005, 0.01) | 33% | | 0.003 (-0.01, 0.01) | 32% | | 0 (-0.009, 0.009) | 0% | |
| Moderate user | -0.001 (-0.01, 0.005) | -13% | | -0.001 (-0.01, 0.01) | -11% | | 0 | 0% | |
| Heavy user | 0.006 (-0.003, 0.02) | 75% | | -0.006 (-0.02, 0.01) | -30% | | 0.007 (-0.007, 0.02) | 67% | |
| **PA (ref High PA)** | |  | |  |  | |  |  | |
| Low PA | 0.002 (-0.005, 0.01) | 20% | | 0.002 (-0.006, 0.01) | 21% | | 0.002 (-0.004, 0.009) | 26% | |
| Moderate PA | 0.003 (-0.002, 0.01) | 33% | | 0.002 (-0.004, 0.008) | 20% | | 0.002 (-0.004, 0.008) | 23% | |
| **Ageing (ref age 40-49)** | |  | |  |  | |  |  | |
| Age 50-59 | 0.005 (0.001, 0.01)** | 81% | | 0.008 (0.001, 0.014)** | 169% | | 0.006 (0.001, 0.01)** | 86% | |
| Age 60-69 | 0.01 (0.001, 0.02)** | 90% | | 0.01 (-0.000, 0.02)* | 93% | | 0.005 (-0.004, 0.01) | 57% | |
| Age 70+ | 0.005 (-0.006, 0.02) | 51% | | 0.002 (-0.01, 0.01) | 12% | | 0.002 (-0.01, 0.01) | 17% | |

* p<0.1; ** p<0.05; *** p<0.01

#### Table S23. The ATT of CHE >40% of total non-food expenditure across different matching algorithms

| **Risk factors** | **CHE >40% of total non-food expenditure** | | | | | | | |  |
| --- | --- | --- | --- | --- | --- | --- | --- | --- | --- |
|  | **Kernel** | | **Nearest Neighbour with replacement** | | | **Nearest Neighbour without replacement** | | |  |
|  | ATT (95% CI) | % relative change to control | | ATT (95% CI) | % relative change to control | | ATT (95% CI) | % relative change to control | |
| **BMI (ref Normal BMI)** | |  | |  |  | |  |  | |
| Overweight | -0.006 (-0.01, 0.001) | -32% | | -0.003 (-0.02, 0.008) | -20% | | -0.004 (-0.01, 0.004) | -24% | |
| Obesity | -0.004 (-0.01, 0.003) | -20% | | -0.007 (-0.01, 0.002) | -28% | | -0.003 (-0.009, 0.004) | -13% | |
| Underweight | 0.004 (-0.005, 0.01) | 22% | | 0.002 (-0.01, 0.02) | 12% | | 0.005 (-0.008, 0.02) | 26% | |
| **Smoking (ref never use)** | |  | |  |  | |  |  | |
| Former user | 0.02 (0.01, 0.04)*** | 160% | | 0.03 (0.01, 0.05)*** | 286% | | 0.02 (0.008, 0.04)*** | 202% | |
| Light user | 0.01 (-0.0003, 0.02)* | 95% | | 0.01 (-0.003, 0.03) | 13% | | 0.009 (-0.004, 0.02) | 90% | |
| Moderate user | 0.002 (-0.005, 0.01) | 21% | | 0 | 0% | | 0.002 (-0.007, 0.01) | 13% | |
| Heavy user | 0.01 (-0.003, 0.02) | 95% | | -0.003 (-0.02, 0.01) | -13% | | 0.01 (-0.006, 0.03) | 120% | |
| **PA (ref High PA)** | |  | |  |  | |  |  | |
| Low PA | 0.006 (-0.002, 0.01) | 40% | | 0.005 (-0.007, 0.02) | 34% | | 0.004 (-0.003, 0.01) | 26% | |
| Moderate PA | 0.003 (-0.003, 0.01) | 23% | | 0.004 (-0.003, 0.01) | 32% | | 0.002 (-0.004, 0.009) | 14% | |
| **Ageing (ref age 40-49)** | |  | |  |  | |  |  | |
| Age 50-59 | 0.01 (0.001, 0.01)** | 59% | | 0.008 (-0.003, 0.018) | 83% | | 0.006 (-0.0009, 0.01)* | 50% | |
| Age 60-69 | 0.01 (0.004, 0.02)*** | 100% | | 0.02 (0.004, 0.03)*** | 125% | | 0.01 (-0.002, 0.02)** | 85% | |
| Age 70+ | 0.015 (0.001, 0.03)** | 115% | | 0.01 (-0.006, 0.03) | 86% | | 0.01 (-0.005, 0.03) | 118% | |

* p<0.1; ** p<0.05; *** p<0.01

#### Table S24. The ATT of labour participation across different matching algorithms

| **Risk factors** | **Labour participation** | | | | | | | |  |
| --- | --- | --- | --- | --- | --- | --- | --- | --- | --- |
|  | **Kernel** | | **Nearest Neighbour with replacement** | | | **Nearest Neighbour without replacement** | | |  |
|  | ATT (95% CI) | % relative change to control | | ATT (95% CI) | % relative change to control | | ATT (95% CI) | % relative change to control | |
| **BMI (ref Normal BMI)** | |  | |  |  | |  |  | |
| Overweight | 0.001 (-0.004, 0.007) | 0% | | 0.03 (0.0004, 0.06)** | 4% | | 0.002 (-0.01, 0.02) | 0% | |
| Obesity | -0.001 (-0.01, 0.01) | 0% | | -0.02 (-0.05, 0.01) | -3% | | 0.0004 (-0.01, 0.02) | 0% | |
| Underweight | 0.003 (-0.006, 0.012) | 0% | | 0.01 (-0.03, 0.04) | 1% | | -0.002 (-0.02, 0.02) | 0% | |
| **Smoking (ref never use)** | |  | |  |  | |  |  | |
| Former user | -0.0003 (-0.02, 0.02) | 0% | | 0.03 (-0.007, 0.07) | 5% | | 0.01 (-0.01, 0.04) | 2% | |
| Light user | 0.005 (-0.008, 0.02) | 1% | | 0.02 (-0.02, 0.05) | 2% | | 0.02 (-0.0003, 0.04)* | 2% | |
| Moderate user | 0.03 (0.02, 0.05)*** | 3% | | 0.02 (-0.002, 0.05)* | 3% | | 0 | 0% | |
| Heavy user | 0.02 (0.003, 0.04)** | 2% | | 0.01 (-0.03, 0.06) | 2% | | 0.002 (-0.02, 0.03) | 0% | |
| **PA (ref High PA)** | |  | |  |  | |  |  | |
| Low PA | -0.005 (-0.01, 0.004) | -1% | | -0.02 (-0.03, -0.002)** | -3% | | 0.006 (-0.003, 0.01) | 1% | |
| Moderate PA | -0.01 (-0.02, -0.003)** | -1% | | -0.007 (-0.03, 0.01) | -1% | | 0.01 (-0.001, 0.02)* | 1% | |
| **Ageing (ref age 40-49)** | |  | |  |  | |  |  | |
| Age 50-59 | 0.0004 (-0.007, 0.01) | 0% | | -0.007 (-0.03, 0.01) | -1% | | 0.01 (-0.001, 0.02)* | 1% | |
| Age 60-69 | -0.02 (-0.04, -0.004)** | -3% | | -0.04 (-0.08, -0.004)** | -6% | | -0.007 (-0.03, 0.02) | -1% | |
| Age 70+ | -0.06 (-0.12, -0.01)** | -12% | | -0.04 (-0.11, 0.04) | -8% | | -0.01 (-0.05, 0.02) | -2% | |

* p<0.1; ** p<0.05; *** p<0.01

#### Table S25. The ATT of the number of days primary activity missed across different matching algorithms

| **Risk factors** | **Number of days primary activity missed** | | | | | | | |  |
| --- | --- | --- | --- | --- | --- | --- | --- | --- | --- |
|  | **Kernel** | | **Nearest Neighbour with replacement** | | | **Nearest Neighbour without replacement** | | |  |
|  | ATT (95% CI) | % relative change to control | | ATT (95% CI) | % relative change to control | | ATT (95% CI) | % relative change to control | |
| **BMI (ref Normal BMI)** | |  | |  |  | |  |  | |
| Overweight | -0.24 (-0.53, 0.06) | -9% | | -0.4 (-0.85, 0.04)* | -13% | | -0.38 (-0.76, -0.006)** | -13% | |
| Obesity | -0.07 (-0.35, 0.20) | -3% | | -0.33 (-0.68, 0.02)* | -11% | | 0.05 (-0.25, 0.35) | 2% | |
| Underweight | 0.36 (-0.13, 0.85) | 11% | | 0.25 (-0.48, 0.97) | 7% | | 0.31 (-0.29, 0.90) | 9% | |
| **Smoking (ref never use)** | |  | |  |  | |  |  | |
| Former user | 1.50 (0.80, 2.18)*** | 52% | | 0.95 (0.20, 1.69)** | 28% | | 1.22 (0.55, 1.90)*** | 41% | |
| Light user | -0.47 (-0.97, 0.04)* | -17% | | -0.48 (-1.28, 0.31) | -17% | | -0.54 (-1.05, -0.04)** | -19% | |
| Moderate user | -0.60 (-1.06, -0.13)** | -22% | | -0.42 (-1.08, 0.25) | -16% | | -0.25 (-0.70, 0.19) | -10% | |
| Heavy user | -0.41 (-1.07, 0.26) | -15% | | -0.46 (-1.47, 0.54) | -17% | | -0.37 (-1.08, 0.34) | -14% | |
| **PA (ref High PA)** | |  | |  |  | |  |  | |
| Low PA | 0.60 (0.2, 0.99)*** | 21% | | 0.58 (-0.02, 1.18)* | 20% | | 0.41 (0.12, 0.70)*** | 17% | |
| Moderate PA | 0.02 (-0.24, 0.27) | 1% | | -0.002 (-0.39, 0.38) | 0% | | 0.03 (-0.24, 0.30) | 1% | |
| **Ageing (ref age 40-49)** | |  | |  |  | |  |  | |
| Age 50-59 | 0.64 (0.40, 0.88)*** | 27% | | 0.63 (0.25, 1.00)*** | 26% | | 0.51 (0.28, 0.73)*** | 23% | |
| Age 60-69 | 0.70 (0.21, 1.19)*** | 24% | | 0.81 (0.12, 1.50)** | 29% | | 0.57 (0.13, 1.01)** | 21% | |
| Age 70+ | 0.18 (-1.1, 1.5) | 4% | | 0.62 (-0.77, 2.01) | 17% | | 0.86 (0.15, 1.58)** | 29% | |

* p<0.1; ** p<0.05; *** p<0.01


#### Table S26. Logistic and ZINB regression for the number of chronic condition and presence of multimorbidity

| **Risk factors** | **Chronic condition** | |
| --- | --- | --- |
|  | **Number of chronic condition** | **Presence of multimorbidity** |
|  | **coef (95% CI)** | **AOR (95% CI)** |
| **Age groups** |  |  |
| 40-49 years | ref | ref |
| 50-59 years | 0.30 (0.25, 0.34)*** | 1.95 (1.72, 2.22)*** |
| 60-69 years | 0.47 (0.42, 0.52)*** | 2.77 (2.39, 3.20)*** |
| 70+ years | 0.54 (0.48, 0.61)*** | 3.02 (2.50, 3.66)*** |
| **BMI group** |  |  |
| Normal BMI (18.5-23.5 kg/m2) | ref | ref |
| Underweight (<18.5 kg/m2) | -0.12 (-0.18, -0.06)*** | 0.97 (0.80, 1.17) |
| Overweight (23.5-24.9 kg/m2) | 0.14 (0.09, 0.19)*** | 1.33 (1.14, 1.55)*** |
| Obesity (>=25 kg/m2) | 0.30 (0.26, 0.34)*** | 1.85 (1.63, 2.10)*** |
| **Physical activity** |  |  |
| High PA | ref | ref |
| Low PA | 0.004 (-0.04, 0.04) | 1.09 (0.96, 1.23) |
| Moderate PA | 0.04 (-0.006, 0.08)* | 1.13 (0.99, 1.29)* |
| **Smoking** |  |  |
| Never smoke | ref | ref |
| Former smoker | 0.26 (0.19, 0.33)*** | 1.78 (1.46, 2.18)*** |
| Light smoker | -0.04 (-0.10, 0.03) | 0.90 (0.72, 1.12) |
| Moderate smoker | -0.11 (-0.17, -0.06)*** | 0.65 (0.54, 0.79)*** |
| Heavy smoker | -0.08 (-0.16, -0.001)** | 0.77 (0.58, 1.02)* |

* p<0.1; ** p<0.05; *** p<0.01

#### Table S27. Logistic and ZINB regression for the number of outpatient and inpatient visits

| **Risk factors** | **Health care utilisation** | | | |
| --- | --- | --- | --- | --- |
|  | Outpatient care | | Inpatient care | |
|  | Process 1 – IRR  IRR (95% CI) | Process 2 – OR  OR (95% CI) | Process 1 - IRR (95% CI) | Process 2 - OR (95% CI) |
| **Age groups** |  |  |  |  |
| 40-49 years | ref | ref | ref | ref |
| 50-59 years | 1.11 (0.94, 1.31) | 0.78 (0.50, 1.22) | 1.51 (1.11, 2.07)** | 6.21 (0.69, 56.13) |
| 60-69 years | 1.18 (0.96, 1.45) | 0.67 (0.39, 1.16) | 1.32 (0.92, 1.90) | 0.58 (0.06, 5.46) |
| 70+ years | 0.92 (0.72, 1.18) | 0.35 (0.16, 0.78)** | 1.16 (0.77, 1.75) | 0.02 (0.00, 3.37) |
| **BMI group** |  |  |  |  |
| Normal BMI (18.5-23.5 kg/m2) | ref | ref | ref | ref |
| Underweight (<18.5 kg/m2) | 1.23 (0.99, 1.52)* | 1.01 (0.60, 1.69) | 1.01 (0.70, 1.45) | 0.22 (0.01, 8.39) |
| Overweight (23.5-24.9 kg/m2) | 1.00 (0.82, 1.22) | 0.95 (0.58, 1.55) | 0.92 (0.65, 1.31) | 0.43 (0.02, 7.74) |
| Obesity (>=25 kg/m2) | 0.92 (0.77, 1.10) | 0.54 (0.32, 0.93)** | 0.96 (0.72, 1.29) | 3.12 (0.58, 16.86) |
| **Physical activity** |  |  |  |  |
| High PA | ref | ref | ref | ref |
| Low PA | 1.20 (1.03, 1.40)** | 1.27 (0.84, 1.92) | 1.42 (1.06, 1.90)** | 1.27 (0.22, 7.36) |
| Moderate PA | 1.05 (0.89, 1.24) | 1.42 (0.90, 2.24) | 1.07 (0.78, 1.47) | 1.90 (0.37, 9.71) |
| **Smoking** |  |  |  |  |
| Never smoke | ref | ref | ref | ref |
| Former smoker | 1.14 (0.88, 1.46) | 0.54 (0.24, 1.23) | 2.04 (1.38, 3.02)*** | 5.67 (0.45, 70.68) |
| Light smoker | 0.77 (0.57, 1.04)* | 1.24 (0.65, 2.39) | 0.57 (0.31, 1.06)* | 48.74 (0.49, 4853.5)* |
| Moderate smoker | 0.60 (0.46, 0.79)*** | 1.01 (0.54, 1.87) | 0.61 (0.39, 0.95)** | 1.76 (0.05, 67.02) |
| Heavy smoker | 0.74 (0.50, 1.11) | 1.14 (0.49, 2.66) | 1.15 (0.58, 2.28) | 135.88 (2.14, 8634.47)** |

* p<0.1; ** p<0.05; *** p<0.01

#### Table S28. Logistic regression for CHE >25% and 40%

| **Risk factors** | **CHE** | |
| --- | --- | --- |
|  | CHE >25% of total household expenditure | CHE >40% of total non-food expenditure |
|  | **AOR (95% CI)** | |
| **Age groups** |  |  |
| 40-49 years | ref | ref |
| 50-59 years | 1.62 (1.01, 2.60)** | 1.49 (1.00, 2.22)* |
| 60-69 years | 2.48 (1.47, 4.17)*** | 2.22 (1.44, 3.44)*** |
| 70+ years | 1.80 (0.86, 3.77) | 2.25 (1.30, 3.89)*** |
| **BMI group** |  |  |
| Normal BMI (18.5-23.5 kg/m2) | ref | ref |
| Underweight (<18.5 kg/m2) | 0.90 (0.47, 1.69) | 1.17 (0.71, 1.92) |
| Overweight (23.5-24.9 kg/m2) | 0.54 (0.28, 1.03)* | 0.75 (0.46, 1.22) |
| Obesity (>=25 kg/m2) | 0.80 (0.50, 1.28) | 0.89 (0.60, 1.31) |
| **Physical activity** |  |  |
| High PA | ref | ref |
| Low PA | 1.69 (1.04, 2.75)** | 1.67 (1.13, 2.48)** |
| Moderate PA | 1.39 (0.84, 2.31) | 1.27 (0.83, 1.95) |
| **Smoking** |  |  |
| Never smoke | ref | ref |
| Former smoker | 2.52 (1.25, 5.09)** | 2.76 (1.63, 4.67)*** |
| Light smoker | 1.88 (0.84, 4.23) | 2.07 (1.10, 3.90)** |
| Moderate smoker | 0.86 (0.38, 1.96) | 1.08 (0.60, 1.95) |
| Heavy smoker | 2.00 (0.81, 4.93) | 2.08 (1.00, 4.30)** |

* p<0.1; ** p<0.05; *** p<0.01

#### Table S29. Logistic and ZINB regression for the productivity loss

| **Risk factors** | **Productivity Loss** | | |
| --- | --- | --- | --- |
|  | Labour participation | Days primary activity missed | |
|  | AOR (95% CI) | Process 1 - IRR (95% CI) | Process 2 - OR (95% CI) |
| **Age groups** |  |  |  |
| 40-49 years | ref | ref | ref |
| 50-59 years | 0.80 (0.70, 0.92)*** | 1.31 (1.21, 1.43)*** | 1.12 (0.99, 1.28)* |
| 60-69 years | 0.32 (0.27, 0.37)*** | 1.38 (1.24, 1.54)*** | 1.13 (0.96, 1.32) |
| 70+ years | 0.12 (0.10, 0.15)*** | 1.45 (1.27, 1.66)*** | 1.24 (1.02, 1.52)** |
| **BMI group** |  |  |  |
| Normal BMI (18.5-23.5 kg/m2) | ref | ref | ref |
| Underweight (<18.5 kg/m2) | 0.78 (0.65, 0.94)** | 1.03 (0.92, 1.16) | 0.89 (0.75, 1.06) |
| Overweight (23.5-24.9 kg/m2) | 1.04 (0.89, 1.23) | 0.94 (0.85, 1.04) | 1.05 (0.90, 1.22) |
| Obesity (>=25 kg/m2) | 0.86 (0.75, 0.98)** | 1.00 (0.91, 1.08) | 1.03 (0.91, 1.17) |
| **Physical activity** |  |  |  |
| High PA | ref | ref | ref |
| Low PA | 0.40 (0.34, 0.46)*** | 1.21 (1.11, 1.32)*** | 1.22 (1.07, 1.38)*** |
| Moderate PA | 0.55 (0.47, 0.64)*** | 0.99 (0.90, 1.08) | 1.04 (0.90, 1.19) |
| **Smoking** |  |  |  |
| Never smoke | ref | ref | ref |
| Former smoker | 0.54 (0.43, 0.68)*** | 1.23 (1.06, 1.42)*** | 0.88 (0.71, 1.10) |
| Light smoker | 1.05 (0.82, 1.33) | 0.95 (0.82, 1.11) | 1.37 (1.11, 1.70)*** |
| Moderate smoker | 1.72 (1.37, 2.17)*** | 0.96 (0.85, 1.09) | 1.49 (1.24, 1.77)*** |
| Heavy smoker | 1.53 (1.09, 2.15)** | 1.04 (0.86, 1.25) | 1.57 (1.23, 2.01)*** |

* p<0.1; ** p<0.05; *** p<0.01
